# An Evidence Map of the Women Veterans’ Health Literature, 2016 to 2023: A Systematic Review

**DOI:** 10.1001/jamanetworkopen.2025.6372

**Published:** 2025-04-22

**Authors:** Karen M. Goldstein, Rachel Pace, Caroline Dancu, Sudha R. Raman, Zoe Bridges-Curry, Patrycja Klimek-Johnson, Athavi Jeevananthan, Anna H. Gallion, Tatyana Der, Amir Alishahi Tabriz, Syketha Sprague, Sharron Rushton, A. Jean Hammer, Catherine A. Sims, Jessica N. Coleman, Justin Martino, Sarah Cantrell, Adelaide M. Gordon, Morgan Jacobs, Anastasia-Stefania Alexopoulos, Dazhe Chen, Jennifer M. Gierisch

**Affiliations:** 1 Durham Center of Innovation to Accelerate Discovery and Practice Transformation, Veterans Affairs (VA) Health Care System, Durham, North Carolina; 2 Department of Medicine, Division of General Internal Medicine, Duke University, Durham, North Carolina; 3 San Francisco VA Health Care System, San Francisco, California; 4 University of California, San Francisco; 5 Department of Population Health Sciences, Duke University School of Medicine, Durham, North Carolina; 6 Duke University Medical Center, Durham, North Carolina; 7 VA Tennessee Valley Healthcare System, Nashville; 8 Vanderbilt University School of Nursing, Nashville, Tennessee; 9 Department of Health Outcomes and Behavior, Moffitt Cancer Center, Tampa, Florida; 10 Department of Medicine—Renal Section, Rocky Mountain Regional VA Medical Center, Aurora, Colorado; 11 Duke University School of Nursing, Durham, North Carolina; 12 University of North Carolina at Chapel Hill School of Nursing, Chapel Hill; 13 Division of Rheumatology, Duke University, Durham, North Carolina; 14 Duke University Medical Center Library and Archives, Duke University School of Medicine, Durham, North Carolina; 15 Epidemiology Branch, National Institute of Environmental Health Sciences, Durham, North Carolina

## Abstract

**Importance:**

Women veterans are the fastest-growing veteran subpopulation in the US. Women veterans often experience military service–related health issues in addition to conditions common to all women. Because women veterans are more likely to receive care in the civilian setting than through the Department of Veterans Affairs (VA), all women’s health clinicians should be equipped to provide patient-centered care for women veterans. The health care of women veterans requires evidence-based care informed by population-specific scientific literature. An updated evidence map evaluating women veteran–focused health literature is needed.

**Objective:**

To map the scope and breadth of women veterans’ health literature published from 2016 to 2023.

**Evidence Review:**

In this systematic review, MEDLINE, Embase, and CINAHL Complete were searched for eligible articles published from 2016 to 2023. Articles reporting about US women veterans’ health outcomes or on the experience of providing care to women veterans were included. Included articles were required to report patient-level outcomes that included either data for only women veterans or reported results separately for women veterans. Articles were grouped by primary focus area based on categories previously established by the VA Women’s Health research agendas and prior evidence maps.

**Findings:**

The volume of women veterans’ health literature published between 2016 and 2023 of 932 articles was double that of the prior 8 years. The largest portion of this literature was focused on chronic medical conditions (137 articles [15%]), general mental health (203 articles [22%]), and interpersonal violence (121 articles 3[13%]). Areas of greatest growth included reproductive health (physical and mental), pain, suicide, and nonsuicidal self-injury. Additionally, emerging areas of inquiry were found, including military-related toxic exposures and harassment within the health care setting.

**Conclusions and Relevance:**

In this systematic review of literature focused on the health of women veterans, the volume of literature was found to have doubled and expanded in important areas that aligned with VA research priorities. However, despite the growth in research related to women veterans, several important research gaps remain within this field of study. Research addressing health issues pertinent to a growing and aging women veterans’ population will require rigorous research and program evaluations.

## Key Points

**Question:**

What is the scope and breadth of women veterans health literature published since 2015?

**Findings:**

This systematic review identified 932 articles published since 2015 about women veterans’ health; most were observational. Overall, the greatest growth was found in literature about reproductive health (physical and mental), pain, suicide, and nonsuicidal self-injury, and there was little growth about long-term care and aging issues.

**Meaning:**

These findings suggest that the women veterans research community has expanded the evidence about issues pertinent to caring for women veterans in areas important to the VA; however, gaps remain around the evaluation of solutions to relevant health problems and on topics pertinent to this aging population.

## Teaser

This systematic review examines the literature on health of women veterans published between 2016 and 2023.

## Introduction

More than 2 million women veterans live in the US.^1^ As the fastest growing veteran group, women veterans represent 10% of the veteran population and are projected to represent 18% by 2040.^1,2^ The number of women veterans who use Veteran Affairs (VA) health care has steadily increased over recent decades, from approximately 160 000 in 2010^3^ to more than 800 000 women veterans as of 2020,^4^ and now includes 28% of all US women veterans.^4^ Additionally, among women veterans using VA health care, 44% receive care services purchased by the VA from community sources.^4^ Thus, women’s health clinicians in VA and non-VA settings must understand the health needs of this unique population to provide patient-centered care. A 2017 evidence map^14^ of women veteran–focused health literature, covering articles published from 2008 to 2015, previously provided a detailed description of available evidence-based research. However, women veterans’ health research has advanced considerably since 2017.

Women veterans face health issues stemming from military service-related experiences, including occupational exposures (eg, combat, toxic and environmental exposures), military sexual trauma (MST), and the physical demands of service (eg, musculoskeletal injuries, intentionally delayed urination).^5,6,7,8^ These experiences can lead directly to chronic health issues, such as posttraumatic stress disorder (PTSD) and chronic pain, and may influence women veteran engagement with the health care system. Many women veterans also have complex multimorbidity comprising of both chronic physical and mental health care challenges and may prefer women clinicians and women-only care environments.^7^ Eighty-six percent of women veterans receiving care in the VA are younger than 65 years and most frequently present with musculoskeletal, mental, reproductive, and endocrine or metabolic-related health issues.^6^ As a population, women veterans are aging. There is a growing proportion of women veterans who are in midlife and require evidence-based treatment for chronic conditions that may be influenced by experiences and exposures that occurred during military service.

Since the 2017 evidence map^14^ of women veterans’ health literature covering publications from 2008 to 2015, women veterans’ health research has advanced considerably. The 2024 White House Initiative on women’s health research highlights the need to advance the evidence base on women veterans health.^9^ A robust understanding of current women veterans health literature is needed to identify where evidence can inform care provision and patient-centered interventions and where additional investigation is needed to optimize care for both VA and civilian women.^5,7,10,11^ Additionally, identification of evidence gaps could inform future exploration to improve the health care of women veterans and civilian women facing similar health challenges. As the associated literature has expanded since the 2017 evidence map, we sought to create an updated evidence map of women veterans’ health literature published since 2015, based on the following question: what is the scope and breadth of the literature on women veterans’ health published since 2015?

## Methods

This systematic review followed the Preferred Reporting Items for Systematic Reviews and Meta-analyses (PRISMA) reporting guideline. Our evidence map was nominated by the VA Office of Women’s Health and conducted by the Durham VA Evidence Synthesis Program. Our scope and organization approach for this literature was informed by input from the nominator and a technical expert panel. A full technical report with detailed methods is available.^12^ The protocol for this map was registered at Open Science Framework.^13^ Our article categorization methodology aligned with the existing approach to women veterans’ health research by the VA Office of Research and Development and Women’s Health Research Network (WHRN).

### Eligibility Criteria

We included peer-reviewed articles that reported US women veterans’ health outcomes or articles reporting on the organization and/or experience of providing care to women veterans. We required that eligible articles reporting patient-level outcomes either included only women veterans’ data or reported results separately for women veterans (eg, subgroup analysis, stratified results). Detailed study eligibility criteria are provided in eTable 1 in Supplement 1.

### Searching and Screening

We searched MEDLINE (via Ovid), Embase (via Elsevier), and CINAHL Complete (via EBSCO) for eligible articles published from January 1, 2016, to October 17, 2023, using a combination of database-specific controlled vocabulary terms and keywords representing women and veterans. Our search strategies were developed in consultation with an expert medical librarian and peer reviewed by a second librarian in accordance with Peer Review of Electronic Search Strategies guidance (eTable 2 in Supplement 1). Two reviewers independently screened all identified citations at the title and abstract level; citations were moved to full text review if included by either reviewer. We reviewed 20% of studies excluded at the title and abstract level to verify accuracy of screening (eg, confirming whether studies with either men and women or women veterans and nonveteran women presented women veterans–specific results). We conducted 3 pilot full-text review rounds of 10 studies each for team calibration prior to independent, single-investigator review. Twenty percent of studies excluded at full-text review by 1 reviewer were reviewed by a second reviewer. If the second reviewer found inconsistency or inaccuracy, they reviewed the remaining citations completed by the first reviewer and corrected as appropriate.

### Data Extraction and Synthesis

A single reviewer extracted key data from all eligible articles. We quality checked data extractions for up to 20% of each reviewer. If accuracy concerns were identified, we reviewed further for accuracy. We also quality-checked all included studies for focus areas and target populations. We assigned a primary focus area to each article based on categories drawn from the prior map.^14^ We modified the prior map’s categories based on input from our technical expert with the goal of adding granularity and flexibility to the linkage of a specific study to a focus area. Specifically, we added new focus areas (eg, toxic exposures) and allowed for each article to be assigned 1 primary focus area and up to 2 secondary focus areas. When multiple potential primary focus areas were identified, we chose the primary focus area based on relevant medical condition and assigned the care delivery characteristic as a secondary focus area. We extracted key study characteristics including study population, focus areas, study design, and funding source. We grouped articles according to focus area and within focus areas, we grouped by specific topics.

### Data Analysis

Descriptive analyses were conducted using Excel version 2402 (Microsoft). Data were analyzed from April to June 2024.

## Results

**Figure 1.**
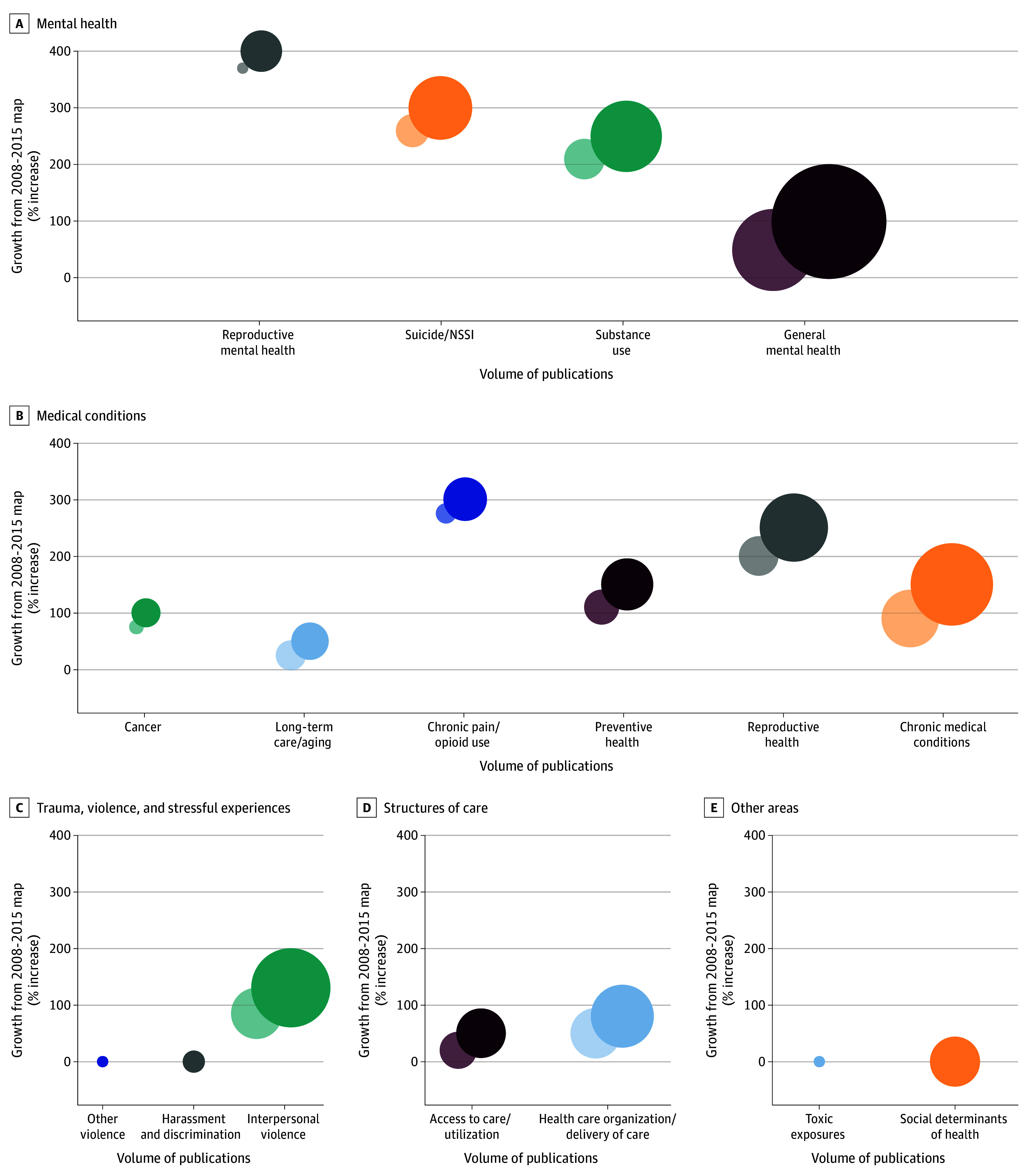
Change in Focus Areas Compared With 2008 to 2015 Evidence Map NSSI indicates nonsuicidal self-injury.

**Figure 2.**
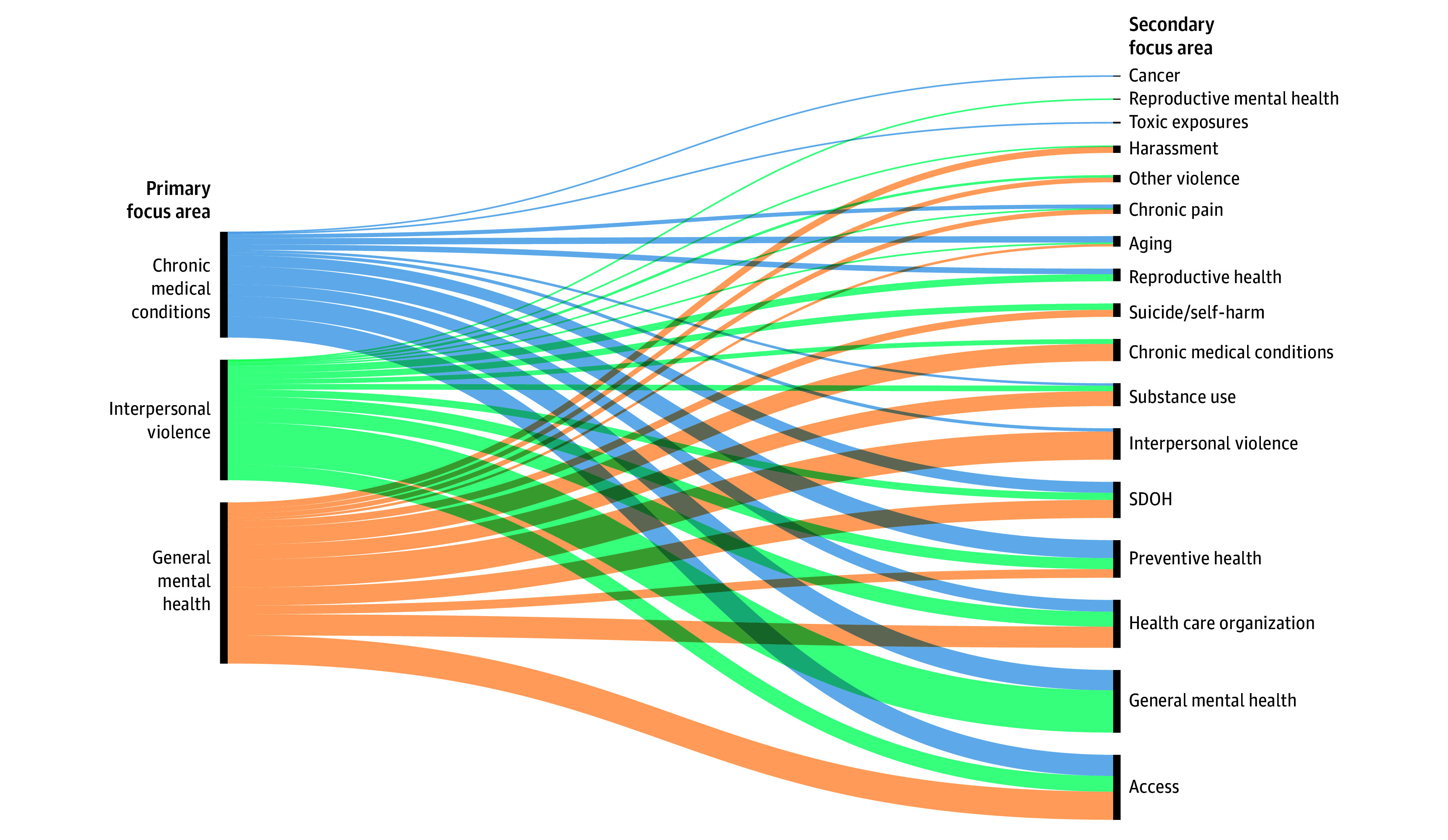
Connection Between Primary and Secondary Focus Areas SDOH indicates social determinants of health.

We identified 932 eligible articles published between 2016 to 2023, representing the work of 598 unique first authors (eFigure 1 in Supplement 1). The technical report provides a full list of included studies and characteristics.^15^ We classified articles to 1 of 16 individual primary focus areas within 5 key categories: mental health (350 articles [38%])^16,17,18,19,20,21,22,23,24,25,26,27,28,29,30,31,32,33,34,35,36,37,38,39,40,41,42,43,44,45,46,47,48,49,50,51,52,53,54,55,56,57,58,59,60,61,62,63,64,65,66,67,68,69,70,71,72,73,74,75,76,77,78,79,80,81,82,83,84,85,86,87,88,89,90,91,92,93,94,95,96,97,98,99,100,101,102,103,104,105,106,107,108,109,110,111,112,113,114,115,116,117,118,119,120,121,122,123,124,125,126,127,128,129,130,131,132,133,134,135,136,137,138,139,140,141,142,143,144,145,146,147,148,149,150,151,152,153,154,155,156,157,158,159,160,161,162,163,164,165,166,167,168,169,170,171,172,173,174,175,176,177,178,179,180,181,182,183,184,185,186,187,188,189,190,191,192,193,194,195,196,197,198,199,200,201,202,203,204,205,206,207,208,209,210,211,212,213,214,215,216,217,218,219,220,221,222,223,224,225,226,227,228,229,230,231,232,233,234,235,236,237,238,239,240,241,242,243,244,245,246,247,248,249,250,251,252,253,254,255,256,257,258,259,260,261,262,263,264,265,266,267,268,269,270,271,272,273,274,275,276,277,278,279,280,281,282,283,284,285,286,287,288,289,290,291,292,293,294,295,296,297,298,299,300,301,302,303,304,305,306,307,308,309,310,311,312,313,314,315,316,317,318,319,320,321,322,323,324,325,326,327,328,329,330,331,332,333,334,335,336,337,338,339,340,341,342,343,344,345,346,347,348,349,350,351,352,353,354,355,356,357,358,359,360,361,362,363,364,365,366,367^; medical conditions (333 articles [36%])^368,369,370,371,372,373,374,375,376,377,378,379,380,381,382,383,384,385,386,387,388,389,390,391,392,393,394,395,396,397,398,399,400,401,402,403,404,405,406,407,408,409,410,411,412,413,414,415,416,417,418,419,420,421,422,423,424,425,426,427,428,429,430,431,432,433,434,435,436,437,438,439,440,441,442,443,444,445,446,447,448,449,450,451,452,453,454,455,456,457,458,459,460,461,462,463,464,465,466,467,468,469,470,471,472,473,474,475,476,477,478,479,480,481,482,483,484,485,486,487,488,489,490,491,492,493,494,495,496,497,498,499,500,501,502,503,504,505,506,507,508,509,510,511,512,513,514,515,516,517,518,519,520,521,522,523,524,525,526,527,528,529,530,531,532,533,534,535,536,537,538,539,540,541,542,543,544,545,546,547,548,549,550,551,552,553,554,555,556,557,558,559,560,561,562,563,564,565,566,567,568,569,570,571,572,573,574,575,576,577,578,579,580,581,582,583,584,585,586,587,588,589,590,591,592,593,594,595,596,597,598,599,600,601,602,603,604,605,606,607,608,609,610,611,612,613,614,615,616,617,618,619,620,621,622,623,624,625,626,627,628,629,630,631,632,633,634,635,636,637,638,639,640,641,642,643,644,645,646,647,648,649,650,651,652,653,654,655,656,657,658,659,660,661,662,663,664,665,666,667,668,669,670,671,672,673,674,675,676,677,678,679,680,681,682,683,684,685,686,687,688,689,690,691,692,693,694,695,696,697,698,699^; structures of care (80 articles [9%])^7,700,701,702,703,704,705,706,707,708,709,710,711,712,713,714,715,716,717,718,719,720,721,722,723,724,725,726,727,728,729,730,731,732,733,734,735,736,737,738,739,740,741,742,743,744,745,746,747,748,749,750,751,752,753,754,755,756,757,758,759,760,761,762,763,764,765,766,767,768,769,770,771,772,773,774,775,776,777,778^; trauma, violence, and stressful experiences (136 articles [15%])^779,780,781,782,783,784,785,786,787,788,789,790,791,792,793,794,795,796,797,798,799,800,801,802,803,804,805,806,807,808,809,810,811,812,813,814,815,816,817,818,819,820,821,822,823,824,825,826,827,828,829,830,831,832,833,834,835,836,837,838,839,840,841,842,843,844,845,846,847,848,849,850,851,852,853,854,855,856,857,858,859,860,861,862,863,864,865,866,867,868,869,870,871,872,873,874,875,876,877,878,879,880,881,882,883,884,885,886,887,888,889,890,891,892,893,894,895,896,897,898,899,900,901,902,903,904,905,906,907,908,909,910,911,912,913,914^; and other focus areas (33 articles [4%]) (Figure 1). The largest primary focus area was general mental health (203 articles [22%]),^166,167,168,169,170,171,172,173,174,175,176,177,178,179,180,181,182,183,184,185,186,187,188,189,190,191,192,193,194,195,196,197,198,199,200,201,202,203,204,205,206,207,208,209,210,211,212,213,214,215,216,217,218,219,220,221,222,223,224,225,226,227,228,229,230,231,232,233,234,235,236,237,238,239,240,241,242,243,244,245,246,247,248,249,250,251,252,253,254,255,256,257,258,259,260,261,262,263,264,265,266,267,268,269,270,271,272,273,274,275,276,277,278,279,280,281,282,283,284,285,286,287,288,289,290,291,292,293,294,295,296,297,298,299,300,301,302,303,304,305,306,307,308,309,310,311,312,313,314,315,316,317,318,319,320,321,322,323,324,325,326,327,328,329,330,331,332,333,334,335,336,337,338,339,340,341,342,343,344,345,346,347,348,349,350,351,352,353,354,355,356,357,358,359,360,361,362,363,364,365,366,367^ followed by chronic medical conditions (137 articles [15%])^564,565,566,567,568,569,570,571,572,573,574,575,576,577,578,579,580,581,582,583,584,585,586,587,588,589,590,591,592,593,594,595,596,597,598,599,600,601,602,603,604,605,606,607,608,609,610,611,612,613,614,615,616,617,618,619,620,621,622,623,624,625,626,627,628,629,630,631,632,633,634,635,636,637,638,639,640,641,642,643,644,645,646,647,648,649,650,651,652,653,654,655,656,657,658,659,660,661,662,663,664,665,666,667,668,669,670,671,672,673,674,675,676,677,678,679,680,681,682,683,684,685,686,687,688,689,690,691,692,693,694,695,696,697,698,699^ and interpersonal violence (121 articles [13%]).^794,795,796,797,798,799,800,801,802,803,804,805,806,807,808,809,810,811,812,813,814,815,816,817,818,819,820,821,822,823,824,825,826,827,828,829,830,831,832,833,834,835,836,837,838,839,840,841,842,843,844,845,846,847,848,849,850,851,852,853,854,855,856,857,858,859,860,861,862,863,864,865,866,867,868,869,870,871,872,873,874,875,876,877,878,879,880,881,882,883,884,885,886,887,888,889,890,891,892,893,894,895,896,897,898,899,900,901,902,903,904,905,906,907,908,909,910,911,912,913,914^ Primary focus areas with the largest proportional increase since the 2008 to 2015 map^14^ were reproductive mental health (5.3-fold), chronic pain or opioid use (4.3-fold), and suicide or nonsuicidal self-injury (4.2-fold). Figure 1 illustrates this growth by circle size for each focus area. Focus areas with the smallest proportional increase were long-term care and aging (1.6-fold) and cancer (2-fold). Most included articles used a descriptive approach with either an observational design (759 of 932 [81%]) or qualitative methods (107 of 932 [12%]). Among the 3 largest primary focus areas, the most common associated secondary focus areas included access to care and use, general mental health, and health care organization or delivery of care for women veterans (Figure 2). Only 32 were experimental (3%), of which 26 were randomized trials^120,137,140,148,170,177,190,198,202,207,220,240,242,251,253,284,361,432,433,575,688,774,804,837,852,910^ (eTable 3 in Supplement 1). We also identified 2 evidence maps,^762,915^ 6 scoping reviews,^156,184,201,380,387,916^ and 9 systematic reviews.^104,247,331,540,684,705,858,872,890^ A summary of included systematic reviews is provided in eTable 4 in Supplement 1.

In this review, 405 of included articles reported on studies using only data for women veterans (44%), while 412 included data from both men and women veterans but reported results separately for women (44%). Additionally, 39 articles focused on data from clinicians, staff, or clinics providing care for women veterans (4%). Finally, 76 articles reported findings in other combined populations (eg, women veterans and civilian women) or in systematic reviews that report number of articles as unit of analysis (7%).

The patient populations most often specified in study eligibility criteria were veterans with service in the Operational Iraqi Freedom (OIF), Operation Enduring Freedom (OEF), and Operation New Dawn (OND) conflicts (156 article [17%]) and 141 veterans with a history of trauma (141 articles [15%]). Compared with the prior 2008 to 2015 map,^4^ there was a notable increase in studies seeking the inclusion of OIF, OEF, or OND (11-fold).

Funding status was reported by most articles (850 [91%]). Multiple funding sources were reported by 210 articles (23%), while 48 articles reported not receiving funding (5%). The most common funding sources were the VA (684 articles [73%]), the National Institute of Health or other government funding (183 articles [20%]), and the US Department of Defense (DOD; 46 articles [5%]). General mental health was the most funded primary focus area for the VA (136 articles [15%]) and DOD (26 articles [3%]). Next, we report results across groups of focus areas assigned to the 5 key categories: mental health; medical conditions; trauma, violence, and stress experiences; structures of care for women veterans; and other focus areas.

### Mental Health

A total of 472 of 932 articles addressed mental health (51%), across 4 primary focus areas: general mental health, substance use, suicide or nonsuicidal self-injury, and reproductive mental health (Table). General mental health was the largest primary focus area overall (203 of 472 articles [43%]),^166,167,168,169,170,171,172,173,174,175,176,177,178,179,180,181,182,183,184,185,186,187,188,189,190,191,192,193,194,195,196,197,198,199,200,201,202,203,204,205,206,207,208,209,210,211,212,213,214,215,216,217,218,219,220,221,222,223,224,225,226,227,228,229,230,231,232,233,234,235,236,237,238,239,240,241,242,243,244,245,246,247,248,249,250,251,252,253,254,255,256,257,258,259,260,261,262,263,264,265,266,267,268,269,270,271,272,273,274,275,276,277,278,279,280,281,282,283,284,285,286,287,288,289,290,291,292,293,294,295,296,297,298,299,300,301,302,303,304,305,306,307,308,309,310,311,312,313,314,315,316,317,318,319,320,321,322,323,324,325,326,327,328,329,330,331,332,333,334,335,336,337,338,339,340,341,342,343,344,345,346,347,348,349,350,351,352,353,354,355,356,357,358,359,360,361,362,363,364,365,366,367^ which included 161 observational studies (79%), 13 RCTs (6%), 10 program evaluations (5%), 2 systematic reviews (1%), and 2 scoping reviews (1%). Topics included in general mental health focus area were: PTSD (95 articles),^167,168,169,170,172,175,181,182,183,187,190,191,194,195,199,202,203,207,210,211,212,213,216,217,219,221,222,224,225,227,229,232,235,239,240,242,243,245,250,253,254,258,260,263,267,268,269,270,271,272,274,275,276,284,285,286,289,290,291,294,296,298,299,300,301,302,303,306,308,314,315,318,319,320,323,327,329,330,332,333,334,336,337,339,341,343,346,347,349,352,353,355,361,362,363^ multiple mental health symptoms or diagnoses (28 articles),^174,176,186,196,209,230,231,241,247,251,252,259,265,266,279,292,293,307,313,316,321,322,345,348,360,364,366^ disordered eating (18 articles),^166,178,179,189,197,204,226,228,237,238,305,309,310,312,335,342,344,365^ sleep-related conditions and symptoms (17 articles),^171,173,177,206,223,248,262,264,273,282,283,287,295,304,311,324,359^ mental health care delivery (17 articles),^184,185,201,205,208,214,220,233,234,244,255,281,288,317,325,350,354^ depression (8 articles),^188,246,249,278,297,328,340,358^ well-being (5 articles),^192,193,198,280,331^ moral injury (3 articles),^200,215,261^ and additional mental health topics (12 articles).^218,236,257,277,326,338,351,356,357,367^ Seventy-one of 203 articles focused on substance use (35%),^95,96,97,98,99,100,101,102,103,104,105,106,107,108,109,110,111,112,113,114,115,116,117,118,119,120,121,122,123,124,125,126,127,128,129,130,131,132,133,134,135,136,137,138,139,140,141,142,143,144,145,146,147,148,149,150,151,152,153,154,155,156,157,158,159,160,161,162,163,164,165^ including 4 RCTs and 1 systematic review. Alcohol and tobacco were most often addressed. Fifty-five of 203 articles focused on suicide or nonsuicidal self-injury (27%),^37,38,39,40,41,42,43,44,45,46,47,48,49,50,51,52,53,54,55,56,57,59,60,61,62,63,64,65,66,67,68,69,70,71,72,73,74,75,76,77,78,80,81,82,83,84,85,86,87,88,89,90,91,92,93^ increasing from 13 in the prior map, in which key topics included prevalence studies, risk factor analyses, formative evaluations (44 articles), VA practices and programs to address suicide (8 articles),^44,45,46,52,53,66,76,81^ and research methods (3 articles).^39,49,74^ Finally, 21 articles addressed reproductive mental health (10%), increasing from 4 in the 2008 to 2015 map, in which peripartum mental health care (9 articles),^16,18,19,21,25,27,29,31,34^ prevalence and risk factors of peripartum mental health (7 articles),^17,20,22,28,30,33,35^ reproductive lifecycle (2 articles),^32,36^ and sexual functioning (3 articles)^23,24,26^ were addressed.

**Table.** Overview of Primary Focus Areas

Primary focus area	Topic or condition	Most common secondary focus areas	Study design	Change since last map (%)
**Mental health**				
General mental health (203 articles)	PTSD (95 articles); multiple mental health symptoms and/or diagnoses (28 articles); disordered eating (18 articles); sleep-related conditions and symptoms (17 articles); mental health care delivery (17 articles); depression (8 articles); well-being (5 articles); moral injury (3 articles); additional mental health topics (12 articles)	Access to care and/or use (36 articles); interpersonal violence (36 articles); health care organization and delivery of care for women veterans (27 articles)	Observational (158 articles); qualitative (16 articles); RCT (13 articles); EPOC and/or quasi-experimental (6 articles); mixed-methods (6 articles); systematic review (4 articles)	109
Substance use (71 articles)	Treatment access, utilization, and outcomes (19 articles); prevalence, associations, and risk factors (17 articles); stress and substance use (15 articles); substance use in marginalized groups (13 articles); screening and detection (7 articles)	General mental health (16 articles); access to care and/or utilization (12 articles); preventative health (9 articles)	Observational (55 articles); qualitative (7 articles); RCT (4 articles); EPOC and/or quasi-experimental (3 articles); systematic review (2 articles)	255
Suicide/NSSI (55 articles)	Prevalence studies, risk factor analyses, and formative evaluations (44 articles); VA practices and programs to address suicide (8 articles); research methods (3 articles)	General mental health (24 articles); SDOH (10 articles); interpersonal violence (9 articles)	Observational (47 articles); qualitative (4 articles); EPOC and/or quasi-experimental (2 articles); mixed-methods (2 articles)	323
Reproductive mental health (21 articles)	Peripartum mental health care (9 articles); prevalence and risk factors of peripartum mental health (7 articles); reproductive lifecycle (2 articles); sexual functioning (3 articles)	Access to care/utilization (7 articles); health care organization and delivery of care for women veterans (7 articles); reproductive health (5 articles); general mental health (4 articles)	Observational (15 articles); qualitative (3 articles); EPOC and/or quasi-experimental (1 article); mixed-methods (2 articles)	425
**Medical conditions**				
Chronic medical conditions (137)	Cardiovascular disorders (32); endocrine disorders (22 articles); nervous system disorders (20 articles); musculoskeletal and rheumatologic disorders (15 articles); military era associated chronic conditions (12 articles); infectious diseases (10 articles); urinary system disorders (10 articles); renal disorders (6 articles); pulmonary disorders (3 articles); gastrointestinal disorders (2 articles); traumatic brain injury (7 articles); amputation (8 articles)	Access to care and/or utilization (26 articles); general mental health (26 articles); preventative health (23 articles)	Observational (128 articles); qualitative (5 articles); RCT (2 articles); mixed-methods (1 article); systematic review (1 article)	132
Reproductive health (88 articles)	Maternal health (30 articles); family planning (29 articles); uterine diagnosis and surgeries (11 articles); menopause (7 articles); sexual health (4 articles); other reproductive health services (7 articles)	Health care organization and delivery of care for women veterans (27 articles); access to care and/or use (27 articles); SDOH (14 articles)	Observational (79 articles); qualitative (5 articles); EPOC and/or quasi-experimental (1 article); mixed-methods (3 articles); systematic review (1 article)	267
Preventative health (46 articles)	Screening for risk factors and/or disease presence (22 articles); health behaviors (22 articles); vaccinations (2 articles)	Health care organization and delivery of care for women veterans (11 articles); general mental health (8 articles); access to care and/or use (8 articles); chronic medical conditions (8 articles)	Observational (36 articles); qualitative (7 articles); RCT (2 articles); mixed-methods (1 article)	156
Chronic pain and opioids (30 articles)	Risk factors for chronic pain (10 articles); opioid use among VA users (8 articles); pain assessment and management (7 articles); health care use among patients with chronic pain (5 articles)	Access to care and/or use (8 articles); health care organization and delivery of care for women veterans (5 articles); general mental health (4 articles)	Observational (27 articles); qualitative (3 articles)	329
Long-term care and aging (21 articles)	Morbidity and mortality (9 articles); cognitive function (7 articles); end-of-life care (3 articles); physical functioning (2 articles)	Chronic medical conditions (6 articles); access to care and/or utilization (3 articles); preventative health (3 articles)	Observational (21 articles); systematic review (2 articles)	62
Cancer (12 articles)	Sex-specific cancers (6 articles); non-sex-specific cancers (6 articles)	Preventative health (3 articles); chronic medical conditions (2 articles); general mental health (1 article)	Observational (11 articles); qualitative (1 article)	100
**Trauma, violence, and stressful experiences**				
Interpersonal violence (121 articles)	Military sexual trauma (69 articles); intimate partner violence (41 articles); sexual violence (5 articles); other interpersonal trauma (6 articles)	General mental health (54 articles); access to care and/or utilization (20 articles); health care organization and delivery of care for women veterans (19 articles)	Observational (86 articles); qualitative (24 articles); RCT (4 articles); EPOC and/or quasi-experimental (2 articles); mixed-methods (3 articles)	163
Other violence (6 articles)	Firearms (4 articles); exposure to violence (2 articles)	General mental health (3 articles); suicide or NSSI (2 articles); interpersonal violence (2 articles)	Observational (4 articles); qualitative (1 article); mixed-methods (1 article)	NA
Harassment and discrimination (9 articles)	Harassment in VA (7 articles); other harassment (2 articles)	Health care organization and delivery of care for women veterans (6 articles); access to care and/or utilization (2 articles); general mental health (1 article); SDOH (1 article)	Observational (5 articles); qualitative (4 articles)	NA
**Structures of care for women veterans**				
Health care organization and delivery of care for women veterans (50 articles)	Service delivery (26 articles); population-specific care needs and preferences (6 articles); staffing and training of VA women’s health care clinicians (7 articles); cost of care (1 article); research methods (10 articles)	Access to care and/or use (11 articles); health care organization and delivery of care for women veterans (9 articles); SDOH (6 articles)	Observational (31 articles); qualitative (15 articles); RCT (1 article); EPOC and/or quasi-experimental (1 article); mixed-methods (2 articles)	61
Access and/or use of care (30 articles)	General access to care and/or utilization (13 articles); specific service access and utilization (4 articles); prioritized population specific utilization and access (12 articles); disability claims (1 article)	Health care organization and delivery of care for women veterans (11 articles); SDOH (7 articles); chronic medical conditions (3 articles)	Observational (21 articles); qualitative (7 articles); mixed-methods (2 articles)	25
**Other focus areas**				
SDOH (30 articles)	Housing instability (15 articles); general or overlapping SDOH (6 articles); other SDOH (6 articles); social outcomes (3 articles)	Access to care and/or use (9 articles); interpersonal violence (7 articles); general mental health (6 articles)	Observational (24 articles); qualitative (4 articles); mixed-methods (2 articles)	NA
Toxic exposures (3 articles)	Toxic exposures (3 articles)	Chronic medical conditions (2 articles); reproductive health (1 article articles); long-term care and aging (1 article articles)	Observational (3 articles)	NA

Abbreviations: EPOC, effective practice and organization of care; NA, not applicable; NSSI, non-suicidal self-injury; PTSD, posttraumatic stress disorder; RCT, randomized clinical trial; SDOH, social determinants of health; VA, Veterans Affairs.

### Medical Conditions

A total of 333 of 932 articles (36%) addressed medical conditions across 6 primary focus areas: chronic medical conditions, reproductive health, preventative health, chronic pain or opioids, long-term care or aging, and cancer (Table). The largest primary focus area was chronic medical conditions (137 of 333 articles [41%]),^564,565,566,567,568,569,570,571,572,573,574,575,576,577,578,579,580,581,582,583,584,585,586,587,588,589,590,591,592,593,594,595,596,597,598,599,600,601,602,603,604,605,606,607,608,609,610,611,612,613,614,615,616,617,618,619,620,621,622,623,624,625,626,627,628,629,630,631,632,633,634,635,636,637,638,639,640,641,642,643,644,645,646,647,648,649,650,651,652,653,654,655,656,657,658,659,660,661,662,663,664,665,666,667,668,669,670,671,672,673,674,675,676,677,678,679,680,681,682,683,684,685,686,687,688,689,690,691,692,693,694,695,696,697,698,699^ of which the most common organ system disorders included cardiovascular (32 articles),^565,574,593,604,609,621,623,625,626,627,629,633,634,636,638,643,645,646,651,654,666,667,671,672,673,680,681,682,683,687,699^ endocrine (eg, obesity, diabetes) (22 articles),^578,582,583,584,586,588,591,594,641,644,648,649,659,668,670,685,688,691,694,696,697,698^ and nervous system (eg, traumatic brain injury) (20 articles).^566,567,568,572,573,576,597,598,599,600,601,612,618,620,642,650,661,663,677,689^ Amputation was addressed by 8 of 333 articles (2%),^564,569,571,577,580,602,605,614^ increasing minorly from 1 article in the prior map. Few articles addressed conditions common to women veterans, such as hypertension, lumbosacral disorders, and irritable bowel syndrome. Reproductive health was addressed in 88 of 333 articles (26%),^476,477,478,479,480,481,482,483,484,485,486,487,488,489,490,491,492,493,494,495,496,497,498,499,500,501,502,503,504,505,506,507,508,509,510,511,512,513,514,515,516,517,518,519,520,521,522,523,524,525,526,527,528,529,530,531,532,533,534,535,536,537,538,539,540,541,542,543,544,545,546,547,548,549,550,551,552,553,554,555,556,557,558,559,560,561,562,563^ increasing from 24 in the last map. The predominant focus in this section was maternal health (30 articles)^477,479,482,483,485,487,488,490,491,493,496,497,501,502,505,506,510,514,516,518,519,523,524,538,541,547,548,549,551,554^ and family planning (29 articles),^478,480,481,489,494,499,503,504,511,512,513,515,517,521,525,527,528,529,533,535,537,542,543,550,552,556,557,558,559^ with others addressing menopause (7 articles)^500,507,509,530,531,546,561^ or sexual health (5 articles).^495,536,544,545,563^ Forty-five of 333 articles (14%) addressed preventive health related issues, including screening for risk factors and/or disease presence (22 articles),^431,432,434,440,441,442,444,445,446,447,449,450,451,452,453,454,457,462,472,474,917^ health behaviors (21 articles)^433,435,438,443,448,455,456,458,459,460,461,463,464,465,466,467,469,470,471,473,475,480^ and vaccinations (2 articles).^436,439^ Thirty of 333 articles addressed chronic pain including opioid use for pain management (9%).^401,402,403,404,405,406,407,408,409,410,411,412,413,414,415,416,417,418,419,420,421,422,423,424,425,426,427,428,429,430^ The smallest primary focus areas in the medical conditions section were long-term care or aging (21 articles [6%])^380,381,382,383,384,385,386,387,388,389,390,391,392,393,394,395,396,397,398,399,400^ and cancer (12 articles [4%]).^401,402,403,404,405,406,407,408,409,410,411,412,413,414,415,416,417,418,419,420,421,422,423,424,425,426,427,428,429,430^ Relative to other sections, long-term care or aging had modest growth from 13 in the 2008 to 2015 map and included topics such as morbidity and mortality (9 articles),^380,386,393,394,395,396,398,400,505^ cognitive function (7 articles),^382,383,385,390,391,392,397^ end-of-life care (3 articles),^381,384,387^ and physical functioning (2 articles).^388,399^ Of the 12 cancer-related articles, 6 addressed breast cancer (or 1.8% of all medical conditions) and 6 addressed nonsex specific cancers (eg, colon cancer or unspecified cancers) (or 1.8% of all medical conditions).

### Trauma, Violence, and Stressful Experiences

A total of 136 of 932 articles addressed trauma, violence, and stressful experiences (15%), across 3 key primary focus areas: interpersonal violence, other violence, and harassment and discrimination (Table). We identified 121 of 136 studies with a primary focus on interpersonal violence (89%), including MST (69 articles),^797,798,799,802,803,805,806,808,810,813,814,815,816,817,818,819,822,823,825,826,828,829,830,833,834,835,836,838,839,842,847,849,851,852,853,854,857,858,862,864,865,868,869,870,871,872,875,877,878,879,882,884,885,886,887,889,892,894,901,903,904,906,907,910,911,912,913,914^ intimate partner violence (41 articles),^794,795,800,801,804,807,811,812,820,821,827,831,832,837,841,843,846,848,850,855,856,859,860,861,866,873,874,876,881,883,888,890,891,893,896,897,898,899,900,908,909^ sexual violence (5 articles),^824,840,844,895,905^ and other interpersonal trauma (6 articles).^796,809,845,867,880,902^ Six of 136 studies used an experimental design (4%), which focused on testing intervention efficacy for MST and intimate partner violence survivors. Nine of 136 articles addressed harassment and discrimination (7%), primarily stranger harassment on VA grounds (6 articles).^786,788,789,790,791,792^ Six of 136 articles addressed other violence types (4%), including firearms (4 articles)^786,788,789,790,791,792^ and exposure to violence, including combat violence (2 articles).^781,784^

### Structures of Care for Women Veterans

The structures of care for women veterans was addressed in 80 of 932 articles (9%), across 2 key primary focus areas: health care organization or delivery of care for women veterans and access or use of care (Table). Of these, 50 articles focused on health care organization and delivery of care for women veterans (63%).^7,730,731,732,733,734,735,736,737,738,739,740,741,742,743,744,745,746,747,748,749,750,751,752,753,754,755,756,757,758,759,760,761,762,763,764,765,766,767,768,769,770,771,772,773,774,775,776,777,778^ Articles in this focus area discussed aspects of models, strategies, staffing, or experiences related to health care organization or delivery of care. Ten articles addressed research methods related to women veterans health were included in this section (13%).^730,734,736,744,746,748,752,761,762,769^ Thirty articles (38%) addressed general access or use of care for women veterans (13 articles),^709,710,712,713,714,715,716,717,718,722,723,727,728^ clinical service-specific articles, such as chiropractic care use (4 articles),^704,708,720,726^ and population-specific articles, such as women veterans with experiences of homelessness (12 articles),^700,701,702,703,705,706,707,719,721,724,725,729^ and disability claims (1 article).^711^ Of note, we identified 166 of 932 articles^7,16,19,20,21,24,31,44,45,53,56,74,100,104,114,116,123,139,153,154,159,160,163,164,170,178,179,184,186,201,204,205,208,210,214,216,233,234,244,251,253,255,260,263,268,269,274,276,283,288,292,294,305,306,316,327,346,354,359,387,393,400,402,403,405,406,414,420,428,438,445,454,455,458,460,475,476,478,481,483,491,492,495,501,502,511,513,517,518,523,524,525,528,529,535,537,539,547,557,559,564,566,567,571,572,577,582,583,584,598,599,600,603,605,619,623,628,642,665,669,682,683,693,697,737,739,742,753,756,757,758,764,766,774,778,791,792,805,812,817,822,834,835,836,844,847,848,852,862,873,876,881,884,886,895,901,904,915,918,919,920,921,922,923,924,925^ that were categorized as having a secondary focus area of access to care (18%) and 153 of 932 articles^16,18,21,26,27,31,34,64,75,105,129,133,140,142,147,154,159,160,179,184,190,199,201,208,224,233,234,250,258,270,272,274,281,288,294,296,311,320,325,330,340,344,347,350,365,377,381,387,404,407,415,427,429,432,438,440,441,444,446,449,457,460,466,471,482,484,485,486,487,491,493,494,499,500,501,502,511,517,522,529,530,531,532,533,538,541,546,548,550,551,553,556,567,568,572,579,580,602,605,619,620,627,628,659,674,676,700,708,709,711,712,713,716,720,723,725,727,730,735,745,756,764,770,772,773,775,784,786,787,788,789,790,792,795,800,802,804,812,819,830,831,832,843,847,856,859,868,870,891,909,910,915,926^ for health organization (16%).

### Other Focus Areas

Thirty of 932 articles (3%) addressed social determinants of health (SDOH),^915,916,918,919,920,921,922,923,924,925,926,927,928,929,930,931,932,933,934,935,936,937,938,939,940,941,942,943,944,945^ and 3 articles focused on toxic exposures (0.3%)^58,79,94^ (Table). Articles on SDOH addressed the influence of nonmedical factors on health outcomes (eg, housing) and key social outcomes not otherwise health-related (eg, employment). All 3 studies on toxic exposure focused on the experiences of Gulf War I Era Veterans.

## Discussion

We found more than 900 articles addressing the women veterans’ health published between 2016 to 2023, almost double that of the preceding 8 years. Similar to the 2008 to 2015 map, the current body of literature remains primarily observational, with a strong focus on mental health. The greatest growth since 2015 was found within the areas of suicide or nonsuicidal self-injury, reproductive mental health, reproductive health, chronic pain or opioids, and interpersonal violence. There was modest growth around long-term care or aging and cancer care for women veterans. Patient populations most often sought for participation were veterans who served in conflicts after September 11, 2001, and those with a history of trauma. Relatively few articles focused on women veterans living in rural areas, despite large numbers of rural women veterans.^6^ We found new collections of articles emerging around harassment and discrimination and military-related toxic exposures.

By design, this evidence map highlights successes in the growth of women veterans health research and identifies directions for future investment. The growth demonstrates the robust evolution of the response to the initial agenda set for 2004 for VA women veterans health research,^946^ which called for priority setting and foster of research based on existing gaps. In fact, the areas of greatest growth largely align with recent priority areas for VA research (eg, pain, opioid use, and suicide prevention) and reflect the successful strategic efforts of the VA WHRN,^947^ including the organization and hosting of relevant national collaborative research workgroups, women veterans health-focused journal supplement sponsorship,^948,949,950^ and direct involvement with congressionally mandated intimate partner violence work.^950,951,952^ Similar strategic efforts will be needed to address the evidence gaps we identified, many pertinent to the shifting demographics of the women veterans population, such as long-term care and aging, cancer, and other chronic medical conditions. Multiple VA efforts are already under way to address these areas, including the recent launch of additional VA WHRN workgroups on menopause, aging, and women’s military exposures, and the release of a special interest notice for VA women’s health topics pertinent to the aging population.^953^ Ongoing effort will be needed to ensure the field remains responsive to the health issues of this dynamic patient population. The VA can support the growth of this literature through incentivizing tracking of enrollment separately for women veterans, encouraging the reporting of results stratified by women or via sex-based analysis when possible, and collaborating with partners, such as the DOD, to expand research activities across the life span of women who serve in the military.

Despite growth in key areas of importance for women veterans health, there has been little shift in research methods along the continuum from descriptive studies to experimental and implementation studies, a need which has been advocated to boost women veterans health research impacts.^14,954^ For example, while we found some increase in trials compared to the 2008 to 2015 map, the overall number was small, and the literature remained predominantly observational. However, we did find a notable number of program evaluations, highlighting the growth of VA clinical offerings and innovations designed to improve the women veterans’ health, and the potential to further leverage the VA’s Learning Health Care System model. Additionally, current efforts to boost the inclusion of women veterans across current and future VA-based trials through the VA’s clinical trials network (VA Cooperative Studies Program) could support the growth of evaluations and testing of women’s health solutions by supporting sex stratified analyses.^949,954^ We identified 411 mixed-sex studies that reported stratified analysis; this approach could be further expanded, as we excluded over 500 articles for a lack of stratified analyses. The growth of diversity, equity, and inclusion efforts across the VA research community may further bolster awareness and commitment to consideration of sex-specific investigations.

Findings from this evidence map have crucial implications beyond the VA.^950^ First, while there are known differences in health status and demographics between VA users and nonusers,^955^ our findings identify strong areas of literature available to support women veterans’ health care, provided in the civilian setting. Second, women veterans are more likely than veteran men to receive VA-purchased care in the community due to a growing need for women’s health expertise and resources beyond what some VA facilities are can support (44% vs 31%).^4^ Thus, clinicians working in both the VA and civilian settings could benefit from a richer understanding of the dynamics and prevalence of health issues and health care challenges experienced by this population. Finally, literature about women veterans includes expertise and knowledge on health issues and complexities that are applicable beyond the veteran population. Comorbid mental and physical health conditions, amputations, SDOH-related care barriers, and long-term consequences of sexual trauma also impact many men and women in the non-veteran civilian population. The extensive expertise built in the VA research community has long supported clinical practice and professional guidelines used by nonveteran civilian populations (eg, shingles vaccine) and women veterans research offers similar benefits.

### Limitations

This review has limitations. First, we aligned our categorization methodology with the existing approach to women veterans’ health research by the VA Office of Research and Development and WHRN. A different approach may have revealed different patterns in the literature. Second, comparison to the prior 2008 to 2015 map may have been inexact due to the subjectivity of focus area assignment. Third, due to the volume of literature, we were unable to screen each citation in duplicate, which may have led to the incorrect exclusion or misclassification of articles. We conducted a limited quality check to identify patterns of error to reduce this risk.

## Conclusions

In this systematic review, the pace of growth of women veterans health research doubled and expanded in important areas that aligned with VA research priorities. A robust evidence base is critical to promote the overall health of women veterans and improve their quality of life and well-being. Further advancement of this field should include research on health issues pertinent to an aging women veterans’ population and greater use of rigorous but pragmatic research and program evaluation approaches.

## Notes

Supplement 1. **eFigure.** Literature Flow Diagram **eTable 1.** Study Eligibility Criteria **eTable 2.** Search Strategies **eTable 3.** Included Trials **eTable 4.** Included Sytematic Reviews
eReferences.

Supplement 2.
Data Sharing Statement
