# Error in Byline

**DOI:** 10.1001/jamanetworkopen.2025.18943

**Published:** 2025-05-23

In the Original Investigation titled “An Evidence Map of the Women Veterans’ Health Literature, 2016 to 2023: A Systematic Review,”^1^ published April 22, 2025, there was a misspelling in Dr Jeevananthan’s last name. This article has been corrected.^1^
