# Supplementary material for: An Evidence Map of the Women Veterans’ Health Literature, 2016 to 2023: A Systematic Review
Source: JAMA Netw Open. 2025 Apr 22;8(4):e256372. doi: 10.1001/jamanetworkopen.2025.6372 (PMC12015682; doi:10.1001/jamanetworkopen.2025.6372)
Supplement: Supplement 1. — eFigure. Literature Flow Diagram eTable 1. Study Eligibility Criteria eTable 2. Search Strategies eTable 3. Included Trials eTable 4. Included Sytematic Reviews eReferences. [file jamanetwopen-e256372-s001.pdf]

Supplemental Online Content

Goldstein KM, Pace R, Dancu C, et al. An evidence map of the women veterans health literature (2016-2023). *JAMA Netw Open*. 2025;8(5):e256372.
doi:10.1001/jamanetworkopen.2025.6372

eFigure. Literature Flow Diagram

eTable 1. Study Eligibility Criteria

eTable 2. Search Strategies

eTable 3. Included Trials

eTable 4. Included Sytematic Reviews

eReferences

This supplemental material has been provided by the authors to give readers additional information about their work.

© 2025 Goldstein KM et al. *JAMA Network Open*.

eFigure 1. Literature flow diagram

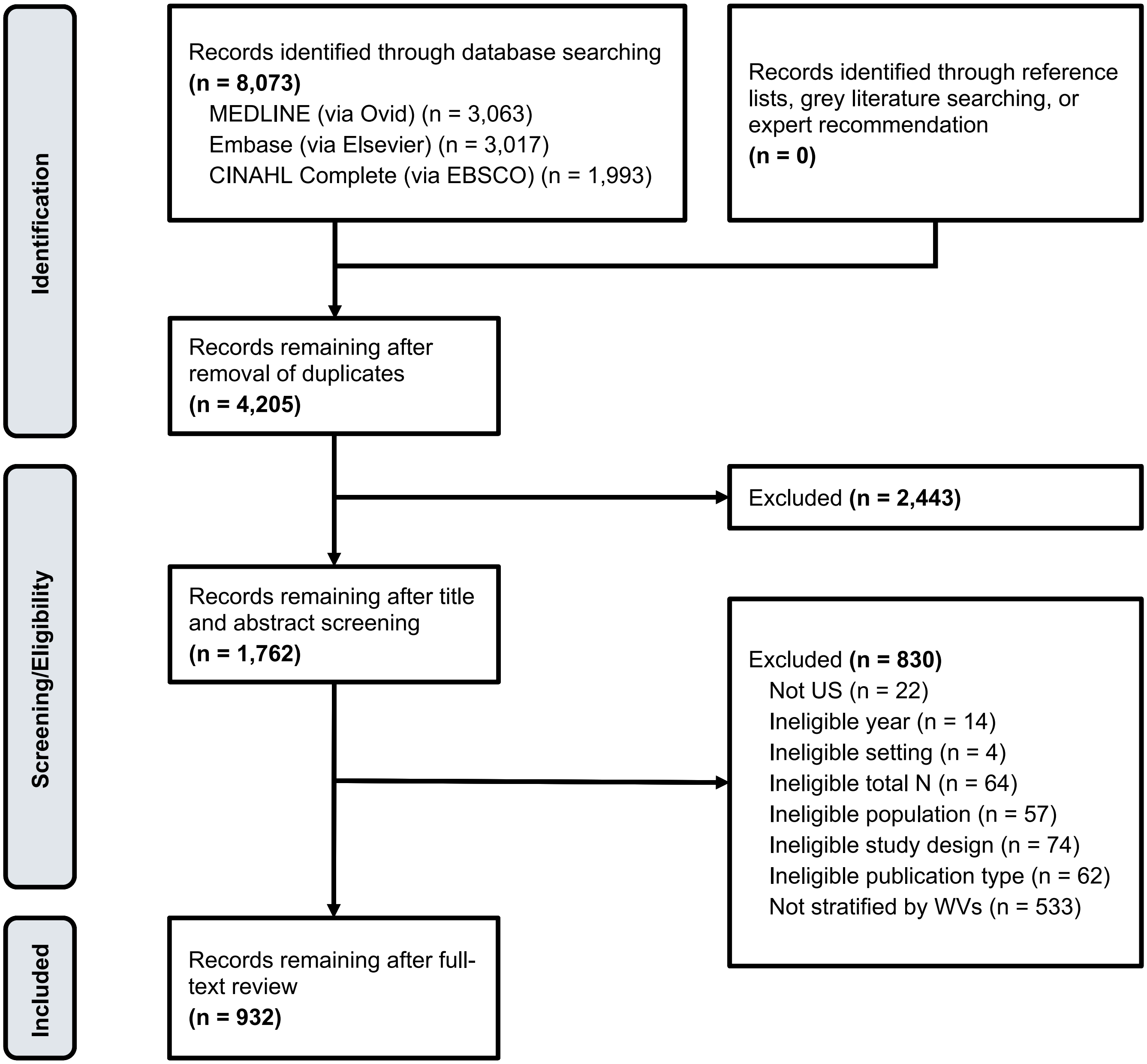

© 2025 Goldstein KM et al. *JAMA Network Open*.

e1. Study eligibility criteria

	Eligibility Criteria
**Population**	Included:<br>• Individuals who have served in the armed forces (including national guard and reserves) and who identify as women or who are transgender and/or non-binary and were assigned female at birth<br>• More than 75% of the study population comprised WVs or the study reported results separately for WVs as subgroup analysis or otherwise reported results separately for women. Included studies could:<br>o Stratify or disaggregate results by sex and/or gender (*e.g.*, report the effect separately for women only)<br>o Report subgroup analysis by sex and/or gender by modeling results separately for men and women<br>o Include mediation modeling or interaction terms to evaluate the contribution of individual sex and/or gender factors to differences between men and women<br>• Healthcare team members who provided care to WVs if the focus of article was on provision of care to the WVs population<br>Excluded:<br>• Studies that did not include US WVs<br>• Studies that included only active-duty members of the military<br>• Animal studies
**Intervention**	Any or none
**Comparator**	Any or none
**Outcomes**	Any
**Setting**	Healthcare settings in the US (or US Veteran expats if outside the US)
**Study design**	Included:<br>• Trials, observational (prospective and retrospective) studies, systematic reviews (*e.g.*, scoping, mapping, umbrella, qualitative), protocols, qualitative studies, secondary analyses of trials, implementation studies, multisite or national program evaluations, measurement or methods studies if specifically used for WVs<br>• Designs other than qualitative or methods development, for which the total number of WVs was over 50<br>• Qualitative studies of only WVs or those with a qualifying subgroup analysis, which included either a specific plan outline to compare men and women or at least 1 theme broken out that was specific to WVs<br>Excluded:<br>• Letters, case reports and case series, meeting abstracts, dissertations not published in a peer reviewed journal, editorials, narrative review, comprehensive or narrative reviews, measurement development studies not specific to WVs, single-site quality improvement projects, commentaries, opinion papers, feasibility studies, pilots<br>• Studies that used sex and/or gender as a component of the regression or propensity model<br>• Studies that treated sex and/or gender as a covariate only
**Years**	Published January 2016 to present
**Language**	English only

© 2025 Goldstein KM et al. *JAMA Network Open*.

eTable 2. Search strategies.

Librarian searcher: Sarah Cantrell, MLIS; Duke University Medical Center Library & Archives, Duke University School of Medicine

Peer review of search conducted by: Samantha Kaplan, PhD, MLIS; Duke University Medical Center Library & Archives, Duke University School of Medicine

Database: MEDLINE (via Ovid)

Search date: 10/17/2023

Note: MEDLINE® ALL 1946 to October 16, 2023

Search Set *Description*	Search Strategy	Results
#1 *Veteran terms*	exp Veterans/ or exp Hospitals, Veterans/ or exp Veterans Health/ or exp Veterans Health Services/ or (veteran or veterans or ((former* or retir*) adj2 (military or "service member" or "service members" or servicemember or servicemembers)) or "post deployment" or post-deployment or VAMC or VAHCS or VHA).ti,ab.	52093
#2 *Women terms*	exp Women/ or exp Women's Health/ or exp Women's Health Services/ or exp Health Services for Transgender Persons/ or exp Homosexuality, Female/ or exp Breast/ or exp Breast Diseases/ or exp Breast Neoplasms/ or exp Mammography/ or exp Mastectomy/ or exp Mammaplasty/ or exp Female Genitalia/ or exp Genital Diseases, Female/ or exp Genital Neoplasms, Female/ or exp Pregnant Women/ or exp Maternal Health/ or exp Maternal Health Services/ or exp Prenatal Care/ or exp Perinatal Care/ or exp Postnatal Care/ or exp Postpartum Period/ or exp Depression, Postpartum/ or exp Pregnancy/ or exp Pregnancy Complications/ or exp Breast Feeding/ or exp Lactation/ or exp Contraception/ or exp Hormonal Contraception/ or exp Contraceptives, Oral/ or exp "Contraceptive Devices, Female"/ or Reproductive Health Services/ or exp Preconception Care/ or exp Family Planning Services/ or exp Fertility/ or exp Infertility/ or exp Fertility Clinics/ or exp obstetrical surgical procedures/ or exp gynecologic surgical procedures/ or exp Abortion, Induced/ or exp Abortion, Spontaneous/ or exp Menstrual Cycle/ or exp Menstruation Disturbances/ or exp Menopause/ or exp Polycystic Ovary Syndrome/ or exp Domestic Violence/ or exp Intimate Partner Violence/ or exp Spousal Abuse/ or exp Battered Women/ or exp Rape/ or (woman or women or womens or womans or "women s" or "woman s" or female or females or "female s" or trans or transgender or transgendered or transfemale or "trans-female" or transman or trans-man or transmans or "transman s" or trans-mans or "trans-man s" or transmen or trans-men or transmens or trans-mens or "transmen s" or "transmen s" or transwoman or trans-woman or transwomans or "transwoman s" or trans-womans or "trans-woman s" or trans-women or transwomen or transwomens or "transwomen s" or trans-womens or "transwomen s" or gender-specific or "gender specific" or gender-related or "gender related" or "gender difference" or "gender differences" or sex-specific or "sex specific" or sex-related or "sex related" or "sex difference" or "sex differences" or lesbian or lesbians or non-binary or "non binary" or abortifacient or abortifacients or abortion or abortions or amenorrhea or breast or breasts or breastfeeding or cervix or cervical or climacteric or clitoris or clitoral or colposcop* or colpotom* or conception or contraception or contraceptive or contraceptives or culdoscop* or dysmenorrhea or dyspareunia or endometriosis or endometritis or endometrium or endometrial or endometrioid or episiotom* or fallopian or fallopians or "family planning" or fertility or gynecolog* or "hot flash" or "hot flashes" or hymen or hymens or hysterectom* or hysteroscop* or infertility or "intimate partner violence" or "intrauterine device" or "intrauterine devices" or IUD or IUDs or labia or labias or labial or lactation or lactating or mammaplast* or mammoplast* or mammogra* or mastectom* or maternal or maternally or menopaus* or menorrhagia or menstrua* or menses or menarche or "military sexual trauma" or "military sexual assault" or "morning after pill" or "morning after pills" or obstetric* or oligomenorrhea or oophorectom* or oophoritis or ovariectom* or ovary or ovaries or ovarian or "painful period" or "painful periods" or "irregular period" or "irregular periods" or PCOS or perimenopaus* or peri-menopaus* or perinatal or peri-natal or perinatally or peri-natally or PMDD or postmenopaus* or post-menopaus* or postnatal or post-natal or postnatally or post-natally or postpartum or post-partum or preconception or pre-conception or pregnancy or pregnancies or pregnant or pregnancy-induced or pregnancy-associated or prepregnancy or premenstrual or pre-menstrual or prenatal or pre-natal or prenatally or pre-natally or puerperium or rape or rapes or raped or "reproductive health" or "reproductive care" or "reproductive healthcare" or "reproductive plan" or "reproductive planning"	4874513

© 2025 Goldstein KM et al. *JAMA Network Open*.

	or salpingectom* or salpingo-oophorectom* or uterus or uterine or vagina or vaginas or vaginal* or transvaginal* or vaginismus or vulva or vulvas or vulvar or vulvectom* or vulvitis or vulvodynia).ti,ab. or ((dilatation or vacuum) adj2 curettage).ti,ab. or ((sex or sexual or sexually or domestic or partner or spouse or spousal or physical or physically) adj3 (abuse or abuses or abused or abuser or abusers or abusive or violence or violent or assault or assaults or assaulted)).ti,ab. or (tubal adj2 (ligation* or sterilization* or sterilisation*)).ti,ab.	
#3<br>*Combination*	1 and 2	5729
#4<br>*Date limit 2016 - present*	Limit 3 to da=20160101-20231231	3156
#5<br>*Study design exclusions*	4 not (case reports OR editorial OR letter OR comment OR congress).pt.	3070
#6<br>*Animal study exclusion*	5 not (exp animals/ not exp humans/)	3063

Database: Embase (via Elsevier)

Search date: 10/17/2023

Note: Search from the Results page

**Search Set** ***Description***	**Search Strategy**	**Results**
#1<br>*Veteran terms*	'veteran'/exp OR 'veterans health'/exp OR 'veterans health service'/exp OR (veteran OR veterans OR ((former* OR retir*) NEAR/2 (military OR 'service member' OR 'service members' OR servicemember OR servicemembers)) OR 'post deployment' OR 'post deployment' OR VAMC OR VAHCS OR VHA):ti,ab	67740
#2<br>*Women terms*	'women`s health'/exp OR 'homosexual female'/exp OR 'male to female transgender'/exp OR 'breast'/exp OR 'breast disease'/exp OR 'breast cancer'/exp OR 'mammography'/exp OR 'mastectomy'/exp OR 'breast reconstruction'/exp OR 'female genital system'/exp OR 'gynecologic disease'/exp OR 'female genital tract tumor'/exp OR 'pregnant woman'/exp OR 'maternal care'/exp OR 'maternal health service'/exp OR 'prenatal care'/exp OR 'perinatal care'/exp OR 'postnatal care'/de OR 'puerperium'/de OR 'postnatal depression'/exp OR 'pregnancy'/exp OR 'pregnancy complication'/exp OR 'breast feeding'/exp OR 'lactation'/exp OR 'lactation disorder'/exp OR 'contraception'/exp OR 'female contraceptive device'/exp OR 'prepregnancy care'/exp OR 'family planning'/exp OR 'female fertility'/exp OR 'female infertility'/exp OR 'fertility clinic'/exp OR 'obstetric procedure'/exp OR 'obstetric operation'/exp OR 'gynecologic disease'/exp OR 'gynecologic surgery'/exp OR 'abortion'/exp OR 'menstrual cycle'/exp OR 'menstrual cycle'/exp OR 'menstruation disorder'/exp OR 'menopause and climacterium'/exp OR 'menopause related disorder'/exp OR 'battered woman'/exp OR 'partner violence'/exp OR 'rape'/exp OR (woman OR women OR womens OR womans OR 'women s' OR 'woman s' OR female OR females OR 'female s' OR trans OR transgender OR transgendered OR transfemale OR 'trans female' OR transman OR 'trans man' OR transmans OR 'transman s' OR 'trans mans' OR 'trans-man s' OR transmen OR 'trans men' OR transmens OR 'trans mens' OR 'transmen s' OR 'transmen s' OR transwoman OR 'trans woman' OR transwomans OR 'transwoman s' OR 'trans womans' OR 'trans-woman s' OR 'trans women' OR transwomen OR transwomens OR 'transwomen s' OR 'trans womens' OR 'transwomen s' OR 'genderspecific' OR 'gender specific' OR 'genderrelated' OR 'gender related' OR 'gender difference' OR 'gender differences' OR 'sexspecific' OR 'sex specific' OR 'sexrelated' OR 'sex related' OR 'sex difference' OR 'sex differences' OR lesbian OR lesbians OR nonbinary OR 'non binary' OR abortifacient OR abortifacients OR abortion OR abortions OR amenorrhea OR breast OR breasts OR breastfeeding OR cervix OR cervical OR climacteric OR clitoris OR clitoral OR colposcop* OR colpotom* OR conception OR contraception OR contraceptive OR contraceptives OR culdoscop* OR dysmenorrhea OR dyspareunia OR endometriosis OR endometritis OR endometrium OR endometrial OR endometrioid OR episiotom* OR fallopian OR fallopians OR 'family planning' OR fertility OR gynecolog* OR 'hot flash' OR 'hot flashes' OR hymen OR hymens OR hysterectom* OR hysteroscop* OR infertility OR 'intimate partner violence' OR 'intrauterine device' OR 'intrauterine devices' OR IUD OR IUDs OR labia OR labias OR labial OR lactation OR lactating OR mammaplast* OR mammoplast* OR mammogra* OR mastectom* OR	7127763

© 2025 Goldstein KM et al. *JAMA Network Open*.

	maternal OR maternally OR menopaus* OR menorrhagia OR menstrua* OR menses OR menarche OR 'military sexual trauma' OR 'military sexual assault' OR 'morning after pill' OR 'morning after pills' OR obstetric* OR oligomenorrhea OR oophorectom* OR oophoritis OR ovariectom* OR ovary OR ovaries OR ovarian OR 'painful period' OR 'painful periods' OR 'irregular period' OR 'irregular periods' OR PCOS OR perimenopaus* OR peri?menopaus* OR perinatal OR peri?natal OR perinatally OR peri?natally OR PMDD OR postmenopaus* OR post?menopaus* OR postnatal OR post?natal OR postnatally OR post?natally OR postpartum OR post?partum OR preconception OR pre?conception OR pregnancy OR pregnancies OR pregnant OR pregnancy?induced OR pregnancy?associated OR prepregnancy OR premenstrual OR pre?menstrual OR prenatal OR pre?natal OR prenatally OR pre?natally OR puerperium OR rape OR rapes OR raped OR 'reproductive health' OR 'reproductive care' OR 'reproductive healthcare' OR 'reproductive plan' OR 'reproductive planning' OR salpingectom* OR salpingo?oophorectom* OR uterus OR uterine OR vagina OR vaginas OR vaginal* OR transvaginal* OR vaginismus OR vulva OR vulvas OR vulvar OR vulvectom* OR vulvitis OR vulvodynia):ti,ab OR ((dilatation OR vacuum) NEAR/2 curettage):ti,ab OR ((sex OR sexual OR sexually OR domestic OR partner OR spouse OR spousal OR physical OR physically) NEAR/3 (abuse OR abuses OR abused OR abuser OR abusers OR abusive OR violence OR violent OR assault OR assaults OR assaulted)):ti,ab OR (tubal NEAR/2 (ligation* OR sterilization* OR sterilisation*)):ti,ab	
#3<br>*Combination*	#1 AND #2	8591
#4<br>*Date limit 2016 - present*	#3 AND [01-01-2016]/sd	5085
#5<br>*Study design exclusions*	#4 NOT ('case report'/exp OR 'case study'/exp OR 'editorial'/exp OR [editorial]/lim OR 'letter'/exp OR [letter]/lim OR 'note'/exp OR [note]/lim OR [conference abstract]/lim OR 'conference abstract'/exp OR 'conference abstract'/it OR 'case report':ti OR editorial:ti OR letter:ti OR proceedings:ti OR conference:ti)	3062
#6<br>*Animal study exclusion*	#5 AND [humans]/lim	3017

Database: CINAHL Complete (via EBSCO)

Search date: 10/17/2023

**Search Set**<br>***Description***	**Search Strategy**	**Results**
#1<br>*Veteran terms*	(MH "Veterans Health Services") OR (MH "Veterans+") OR (MH "United States Department of Veterans Affairs") OR (MH "Hospitals, Veterans") OR ((TI veteran OR AB veteran) OR (TI veterans OR AB veterans) OR (((TI former* OR AB former*) OR (TI retir* OR AB retir*)) N2 ((TI military OR AB military) OR (TI "service member" OR AB "service member") OR (TI "service members" OR AB "service members") OR (TI servicemember OR AB servicemember) OR (TI servicemembers OR AB servicemembers))) OR (TI "post deployment" OR AB "post deployment") OR (TI post-deployment OR AB post-deployment) OR (TI VAMC OR AB VAMC) OR (TI VAHCS OR AB VAHCS) OR (TI VHA OR AB VHA))	37061
#2<br>*Women terms*	(MH "Women+") OR (MH "Battered Women") OR (MH "Single Women") OR (MH "Trans Women") OR (MH "Expectant Mothers") OR (MH "Intimate Partner Violence") OR (MH "Women's Health") OR (MH "Women's Health Services") OR (MH "Lesbians") OR (MH "Breast+") OR (MH "Breast Neoplasms+") OR (MH "Breast Self-Examination") OR (MH "Breast Pumps") OR (MH "Breast Tissue Density") OR (MH "Breast Reconstruction") OR (MH "Breast Examination+") OR (MH "Breast Implants") OR (MH "Breast Feeding+") OR (MH "Mammography") OR (MH "Mastectomy+") OR (MH "Genitalia, Female+") OR (MH "Genital Neoplasms, Female+") OR (MH "Genital Diseases, Female+") OR (MH "Female Urogenital Diseases and Pregnancy Complications+") OR (MH "Sexual Dysfunction, Female+") OR (MH "Maternal Health Services+") OR (MH "Prenatal Care") OR (MH "Prenatal Diagnosis+") OR (MH "Perinatal Period") OR (MH "Perinatal Care") OR (MH "Postnatal Care+") OR (MH "Postnatal Period+") OR (MH "Depression, Postpartum") OR (MH "Postpartum Psychosis") OR (MH "Pregnancy+") OR (MH "Pregnancy Outcomes") OR (MH "Pregnancy Discomforts") OR (MH "Lactation") OR (MH "Lactation Disorders+") OR (MH "Contraception+") OR (MH "Hormonal Contraception") OR (MH "Contraceptive Agents+") OR (MH "Prepregnancy Care") OR (MH "Family Planning+") OR (MH "Fertility+") OR (MH "Fertility Preservation") OR (MH "Infertility+") OR (MH "Obstetric Emergencies") OR (MH "Delivery, Obstetric+")	1459233

© 2025 Goldstein KM et al. *JAMA Network Open*.

	OR (MH "Obstetric Patients") OR (MH "Obstetric Service") OR (MH "Obstetric Care+") OR (MH "Surgery, Obstetrical+") OR (MH "Gynecologic Examination") OR (MH "Surgery, Gynecologic+") OR (MH "Diagnosis, Gynecologic+") OR (MH "Gynecologic Care") OR (MH "Abortion, Incomplete") OR (MH "Abortion, Induced+") OR (MH "Abortion, Spontaneous+") OR (MH "Menstrual Cycle+") OR (MH "Dysmenorrhea") OR (MH "Menstrual and Perimenopausal Disorders+") OR (MH "Menstruation Disorders+") OR (MH "Menstruation") OR (MH "Menstruation Inducing Agents+") OR (MH "Oligomenorrhea") OR (MH "Menopause+") OR (MH "Menarche") OR (MH "Menopause, Premature") OR (MH "Premenopause") OR (MH "Postmenopause") OR (MH "Postmenopausal Disorders") OR (MH "Perimenopause") OR (MH "Domestic Violence+") OR (MH "Rape") OR ((TI woman OR AB woman) OR (TI women OR AB women) OR (TI womens OR AB womens) OR (TI womans OR AB womans) OR (TI "women s" OR AB "women s") OR (TI "woman s" OR AB "woman s") OR (TI female OR AB female) OR (TI females OR AB females) OR (TI "female s" OR AB "female s") OR (TI trans OR AB trans) OR (TI transgender OR AB transgender) OR (TI transgendered OR AB transgendered) OR (TI transfemale OR AB transfemale) OR (TI trans-female OR AB trans-female) OR (TI transman OR AB transman) OR (TI trans-man OR AB trans-man) OR (TI transmans OR AB transmans) OR (TI "transman s" OR AB "transman s") OR (TI trans-mans OR AB trans-mans) OR (TI "trans-man s" OR AB "trans-man s") OR (TI transmen OR AB transmen) OR (TI trans-men OR AB trans-men) OR (TI transmens OR AB transmens) OR (TI trans-mens OR AB trans-mens) OR (TI "transmen s" OR AB "transmen s") OR (TI "transmen s" OR AB "transmen s") OR (TI transwoman OR AB transwoman) OR (TI trans-woman OR AB trans-woman) OR (TI transwomans OR AB transwomans) OR (TI "transwoman s" OR AB "transwoman s") OR (TI trans-womans OR AB trans-womans) OR (TI "trans-woman s" OR AB "trans-woman s") OR (TI trans-women OR AB trans-women) OR (TI transwomen OR AB transwomen) OR (TI transwomens OR AB transwomens) OR (TI "transwomen s" OR AB "transwomen s") OR (TI trans-womens OR AB trans-womens) OR (TI "transwomen s" OR AB "transwomen s") OR (TI gender-specific OR AB gender-specific) OR (TI "gender specific" OR AB "gender specific") OR (TI gender-related OR AB gender-related) OR (TI "gender related" OR AB "gender related") OR (TI "gender difference" OR AB "gender difference") OR (TI "gender differences" OR AB "gender differences") OR (TI sex-specific OR AB sex-specific) OR (TI "sex specific" OR AB "sex specific") OR (TI sex-related OR AB sex-related) OR (TI "sex related" OR AB "sex related") OR (TI "sex difference" OR AB "sex difference") OR (TI "sex differences" OR AB "sex differences") OR (TI lesbian OR AB lesbian) OR (TI lesbians OR AB lesbians) OR (TI non-binary OR AB non-binary) OR (TI "non binary" OR AB "non binary") OR (TI abortifacient OR AB abortifacient) OR (TI abortifacients OR AB abortifacients) OR (TI abortion OR AB abortion) OR (TI abortions OR AB abortions) OR (TI amenorrhea OR AB amenorrhea) OR (TI breast OR AB breast) OR (TI breasts OR AB breasts) OR (TI breastfeeding OR AB breastfeeding) OR (TI cervix OR AB cervix) OR (TI cervical OR AB cervical) OR (TI climacteric OR AB climacteric) OR (TI clitoris OR AB clitoris) OR (TI clitoral OR AB clitoral) OR (TI colposcop* OR AB colposcop*) OR (TI colpotom* OR AB colpotom*) OR (TI conception OR AB conception) OR (TI contraception OR AB contraception) OR (TI contraceptive OR AB contraceptive) OR (TI contraceptives OR AB contraceptives) OR (TI culdoscop* OR AB culdoscop*) OR (TI dysmenorrhea OR AB dysmenorrhea) OR (TI dyspareunia OR AB dyspareunia) OR (TI endometriosis OR AB endometriosis) OR (TI endometritis OR AB endometritis) OR (TI endometrium OR AB endometrium) OR (TI endometrial OR AB endometrial) OR (TI endometrioid OR AB endometrioid) OR (TI episiotom* OR AB episiotom*) OR (TI fallopian OR AB fallopian) OR (TI fallopians OR AB fallopians) OR (TI "family planning" OR AB "family planning") OR (TI fertility OR AB fertility) OR (TI gynecolog* OR AB gynecolog*) OR (TI "hot flash" OR AB "hot flash") OR (TI "hot flashes" OR AB "hot flashes") OR (TI hymen OR AB hymen) OR (TI hymens OR AB hymens) OR (TI hysterectom* OR AB hysterectom*) OR (TI hysteroscop* OR AB hysteroscop*) OR (TI infertility OR AB infertility) OR (TI "intimate partner violence" OR AB "intimate partner violence") OR (TI "intrauterine device" OR AB "intrauterine device") OR (TI "intrauterine devices" OR AB "intrauterine devices") OR (TI IUD OR AB IUD) OR (TI IUDs OR AB IUDs) OR (TI labia OR AB labia) OR (TI labias OR AB labias) OR (TI labial OR AB labial) OR (TI lactation OR AB lactation) OR (TI lactating OR AB lactating) OR (TI mammaplast* OR AB mammaplast*) OR (TI mammoplast* OR AB mammoplast*) OR (TI mammogra* OR AB mammogra*) OR (TI mastectom* OR AB mastectom*) OR (TI maternal OR AB maternal) OR (TI maternally OR AB maternally) OR (TI menopaus* OR AB menopaus*) OR (TI menorrhagia OR AB menorrhagia) OR (TI menstrua* OR AB menstrua*) OR (TI menses OR AB menses) OR (TI menarche OR AB menarche) OR (TI "military sexual trauma" OR AB "military sexual trauma") OR (TI "military sexual assault" OR AB "military sexual assault") OR (TI "morning after pill" OR AB "morning after pill") OR (TI "morning after pills" OR AB "morning after pills") OR (TI obstetric* OR AB obstetric*) OR (TI oligomenorrhea OR AB oligomenorrhea) OR (TI oophorectom* OR AB oophorectom*) OR (TI oophoritis OR AB oophoritis) OR (TI ovariectom* OR AB ovariectom*) OR (TI ovary OR AB ovary) OR (TI ovaries OR AB ovaries) OR (TI ovarian OR AB ovarian) OR (TI "painful period" OR AB "painful period") OR (TI "painful periods" OR AB "painful periods") OR (TI "irregular period" OR AB "irregular period") OR (TI "irregular periods" OR AB "irregular periods") OR (TI PCOS OR AB PCOS) OR (TI perimenopaus* OR AB perimenopaus*) OR (TI peri-menopaus* OR AB peri-menopaus*) OR (TI perinatal OR AB perinatal) OR (TI peri-natal OR AB peri-natal) OR (TI perinatally OR AB perinatally) OR (TI peri-natally OR AB peri-natally) OR (TI PMDD OR AB PMDD) OR (TI postmenopaus* OR AB postmenopaus*) OR (TI post-menopaus* OR AB post-menopaus*) OR (TI postnatal OR AB postnatal) OR (TI post-natal OR AB post-natal) OR (TI postnatally OR AB postnatally) OR (TI post-natally OR AB post-natally) OR (TI postpartum OR AB postpartum) OR (TI post-partum OR AB post-partum) OR (TI preconception OR AB preconception) OR (TI pre-conception OR AB pre-conception) OR (TI pregnancy OR AB pregnancy) OR (TI pregnancies OR AB pregnancies) OR (TI pregnant OR AB pregnant) OR (TI pregnancy-induced OR AB pregnancy-induced) OR (TI pregnancy-associated OR AB pregnancy-associated)	

	OR (TI prepregnancy OR AB prepregnancy) OR (TI premenstrual OR AB premenstrual) OR (TI pre-menstrual OR AB pre-menstrual) OR (TI prenatal OR AB prenatal) OR (TI pre-natal OR AB pre-natal) OR (TI prenatally OR AB prenatally) OR (TI pre-natally OR AB pre-natally) OR (TI puerperium OR AB puerperium) OR (TI rape OR AB rape) OR (TI rapes OR AB rapes) OR (TI raped OR AB raped) OR (TI "reproductive health" OR AB "reproductive health") OR (TI "reproductive care" OR AB "reproductive care") OR (TI "reproductive healthcare" OR AB "reproductive healthcare") OR (TI "reproductive plan" OR AB "reproductive plan") OR (TI "reproductive planning" OR AB "reproductive planning") OR (TI salpingectom* OR AB salpingectom*) OR (TI salpingo-oophorectom* OR AB salpingo-oophorectom*) OR (TI uterus OR AB uterus) OR (TI uterine OR AB uterine) OR (TI vagina OR AB vagina) OR (TI vaginas OR AB vaginas) OR (TI vaginal* OR AB vaginal*) OR (TI transvaginal* OR AB transvaginal*) OR (TI vaginismus OR AB vaginismus) OR (TI vulva OR AB vulva) OR (TI vulvas OR AB vulvas) OR (TI vulvar OR AB vulvar) OR (TI vulvectom* OR AB vulvectom*) OR (TI vulvitis OR AB vulvitis) OR (TI vulvodynia OR AB vulvodynia)) OR (((TI dilatation OR AB dilatation) OR (TI vacuum OR AB vacuum)) N2 (TI curettage OR AB curettage)) OR (((TI sex OR AB sex) OR (TI sexual OR AB sexual) OR (TI sexually OR AB sexually) OR (TI domestic OR AB domestic) OR (TI partner OR AB partner) OR (TI spouse OR AB spouse) OR (TI spousal OR AB spousal) OR (TI physical OR AB physical) OR (TI physically OR AB physically)) N3 ((TI abuse OR AB abuse) OR (TI abuses OR AB abuses) OR (TI abused OR AB abused) OR (TI abuser OR AB abuser) OR (TI abusers OR AB abusers) OR (TI abusive OR AB abusive) OR (TI violence OR AB violence) OR (TI violent OR AB violent) OR (TI assault OR AB assault) OR (TI assaults OR AB assaults) OR (TI assaulted OR AB assaulted))) OR ((TI tubal OR AB tubal) N2 ((TI ligation* OR AB ligation*) OR (TI sterilization* OR AB sterilization*) OR (TI sterilisation* OR AB sterilisation*)))	
#3<br>*Combination*	S1 AND S2	4720
#4<br>*Date limit 2016 - present*	Limit 3 to da=20160101-20231231	2253
#5<br>*Study design exclusions*	4 not (case reports OR editorial OR letter OR comment OR congress).pt.	1996
#6<br>*Animal study exclusion*	5 not (exp animals/ not exp humans/)	1993

© 2025 Goldstein KM et al. *JAMA Network Open*.

eTable 3. Included Trials

Author, Year<br>N (% women)	Title	Objective	Secondary focus areas	Target populations<br><br>Funding
**General mental health**				
Acierno, 2021[1]<br>136 (100%)	A Randomized Clinical Trial of In-person vs. Home-based Telemedicine Delivery of Prolonged Exposure for PTSD in Military Sexual Trauma Survivors	This study used a randomized controlled design to examine PTSD and<br>depression symptom outcomes, overall number of sessions completed	Access to care/utilization	History of trauma<br><br>DOD
Amsalem, 2021[2]<br>172 (31.39%)	Increasing Treatment-Seeking Intentions of US Veterans in the Covid-19 era: A Randomized Controlled Trial	This study screened for clinical symptoms and evaluated the efficacy of a brief, online social contact based video intervention in increasing treatment seeking intentions among veterans	Access to care/utilization	N/A<br><br>Not reported
Castillo, 2016[3]<br>86 (100%)	Group-Delivered Cognitive/Exposure Therapy for PTSD in Women Veterans: A Randomized Controlled Trial	The primary aim of this RCT was to examine the overall efficacy of the 16-week, three-module group protocol on PTSD severity compared to a minimal attention waitlist control in a sample of Afghanistan (OEF) and Iraq (OIF) WVs	N/A	History of trauma; OEF/OIF/OND<br><br>DOD
Creech, 2022[4]<br>153 (100%)	Computerized Intervention in Primary Care for Women Veterans with Sexual Assault Histories and Psychosocial Health Risks: a Randomized Clinical Trial	This study aimed to test the impact of a brief computerized intervention delivered in primary care to reduce health risks and increase mental health treatment utilization among women with histories of sexual assault and current health risks	Substance use<br><br>General mental health	History of trauma<br><br>NIH/other government
Gobin, 2019[5]<br>160 (20%)	Gender Differences in Response to Acceptance and Commitment Therapy Among Operation Enduring Freedom/Operation Iraqi Freedom/Operation New Dawn Veterans	The purpose of this investigation was to examine gender differences in response to acceptance and commitment therapy, an empirically supported transdiagnostic psychotherapy	N/A	OEF/OIF/OND<br><br>DOD
Kelly, 2021[6]<br>104 (100%)	Trauma-Sensitive Yoga for Post-Traumatic Stress Disorder in Women Veterans who Experienced Military Sexual Trauma: Interim Results from a Randomized Controlled Trial	This study aimed to assess the effectiveness of Trauma Center Trauma-Sensitive Yoga for PTSD among WVs with PTSD related to MST	Interpersonal violence	History of trauma<br><br>VA
Lehavot, 2021[7]<br>102 (100%)	A Randomized Trial of an Online, Coach-Assisted Self-Management PTSD Intervention Tailored for Women Veterans	This RCT aimed to evaluate the feasibility, acceptability, and efficacy of DElivery of Self TRaining and Education for Stressful Situations (DESTRESS)-WV compared to an active control (phone monitoring) on PTSD symptoms at post-treatment and at three- and six-months post-treatment	N/A	N/A<br><br>Not reported
Lopez, 2022[8]<br>151 (100%)	Effects of Emotion Dysregulation on Post-treatment Post-traumatic Stress Disorder and Depressive Symptoms Among Women Veterans With Military Sexual Trauma	This study aimed to present secondary data analyses from a randomized clinical trial comparing the efficacy of in-person versus telemedicine delivery of prolonged exposure therapy for WVs with MST-related PTSD	Interpersonal violence	History of trauma<br><br>NIH/other government; DOD

© 2025 Goldstein KM et al. *JAMA Network Open*.

Author, Year<br>N (% women)	Title	Objective	Secondary focus areas	Target populations<br><br>Funding
Martin, 2023[9]<br>149 (100%)	Novel Treatment Based on Acceptance and Commitment Therapy Versus Cognitive Behavioral Therapy for Insomnia: A Randomized Comparative Effectiveness Trial in Women Veterans	The primary objectives of this study were to determine whether acceptance and commitment therapy for insomnia (ABC-I) was noninferior to cognitive behavioral therapy for insomnia (CBT-I) in improving sleep and to test whether ABC-I resulted in higher treatment completion and adherence versus CBT-I.	N/A	NA<br><br>VA; NIH/other government
Murdoch, 2022[10]<br>383 (50.30%)	Impact of Different Cover Letter Information and Incentives on Veterans' Emotional Responses to an Unsolicited Mailed Survey About Military Traumas: A Randomized, 3x2x2 Factorial Trial	This postal survey study aimed to test whether altering cover letter information would induce or reduce non-response bias in a sample of US Veterans applying for PTSD disability benefits, and examined whether those cover letter alterations impacted participant emotional reactions	N/A	OEF/OIF/OND<br><br>VA
Saban, 2022[11]<br>136 (100%)	Impact of a Mindfulness-Based Stress Reduction Program on Psychological Well-Being, Cortisol, and Inflammation in Women Veterans	This study determined the effectiveness of the Mindfulness-Based Stress Reduction Program for improving psychological well-being, cortisol, and inflammation associated with CVD in WVs.	Preventative health<br>Chronic medical conditions	N/A<br><br>VA
Thompson-Hollands, 2023[12]<br>284 (100%)	Treatment Length and Symptom Improvement in Prolonged Exposure and Present-Centered Therapy for Posttraumatic Stress Disorder: Comparing Dose-Response and Good-Enough Level Models in Two Manualized Interventions	To compare two theories (the dose-response model and the good-enough level model) across two manualized treatments for PTSD	Access to care/utilization	History of trauma<br><br>DOD
Zaccari, 2022[13]<br>152 (100%)	Synchronous Telehealth Yoga and Cognitive Processing Group Therapies for Women Veterans with Posttraumatic Stress Disorder: A Multisite Randomized Controlled Trial Adapted for COVID-19	This study aimed to present modifications made to pivot a multisite RCT at a Southeastern and Pacific Northwestern VA Health Care Systems from in-person to virtual study implementation.	Health care organization/ delivery of care for WV<br><br>Interpersonal violence	History of trauma<br><br>VA
Substance Use				
Danan, 2019[14]<br>2654 (5.22%)	Smoking Cessation among Female and Male Veterans before and after a Randomized Trial of Proactive Outreach	This study compared baseline experiences with VA smoking cessation care for men and women and assessed differing gender responses to a proactive intervention	N/A	N/A<br><br>VA
Holzhauer, 2021[15]<br>50 (100%)	Targeting Women Veteran's Stress-Induced Drinking with Cognitive Reappraisal: Mechanisms and Moderators of Change	This study examined the effects of cognitive reappraisal, an adaptive emotion regulation strategy, on mechanisms that contribute to drinking among 50 WVs.	General mental health	N/A<br><br>VA
Najavits, 2018[16]<br>66 (100%)	A Randomized Controlled Trial of a Gender-Focused Addiction Model Versus 12-Step Facilitation for Women Veterans	This study compared 12 individual sessions of a gender-focused substance use disorder (SUD) recovery model, 'A Womans Path to Recovery' to an evidence-based, non-gender-focused SUD model, 12-Step Facilitation, in WVs with severe SUD	Health care organization/delivery of care for WV	Justice-involved<br><br>VA
Pedersen, 2017[17]<br>784 (17%)	A Randomized Controlled Trial of a Web-Based, Personalized Normative Feedback Alcohol Intervention for Young-Adult Veterans	This RCT tested a very brief alcohol intervention delivered over the Internet to reach the population of young adult veterans to help reduce their drinking	Preventative health	N/A<br><br>NIH/other government
Interpersonal Violence				

© 2025 Goldstein KM et al. *JAMA Network Open*.

Author, Year<br>N (% women)	Title	Objective	Secondary focus areas	Target populations<br><br>Funding
Gilmore, 2016[18]<br>100 (100%)	"Do You Expect Me to Receive PTSD Care in a Setting Where Most of the Other Patients Remind Me of the Perpetrator?": Home-Based Telemedicine to Address Barriers to Care Unique to Military Sexual Trauma and Veterans Affairs Hospitals	This manuscript described an ongoing federally funded RCT comparing PE delivered in-person to PE delivered via home-based telemedicine	Health care organization/delivery of care for WV<br><br>General mental health	History of trauma<br><br>NIH/other government
Gilmore, 2020[19]<br>136 (100%)	Emotion Dysregulation Predicts Dropout from Prolonged Exposure Treatment among Women Veterans with Military Sexual Trauma-Related Posttraumatic Stress Disorder	This study examined factors associated with treatment dropout among WVs with MST-related PTSD enrolled in PE both in person or via telemedicine as part of an ongoing randomized clinical trial	Access to care/utilization<br><br>General mental health	History of trauma<br><br>NIH/other government; DOD
Iverson, 2021[20]<br>60 (100%)	Recovering From Intimate Partner Violence Through Strengths and Empowerment: Findings From a Randomized Clinical Trial	This study examined the effectiveness of RISE compared to an advocacy-based enhanced care as usual condition consisting of education, safety planning, resources, and referrals in a sample of women VHA patients.	N/A	History of trauma<br><br>VA
Iverson, 2023[21]<br>7421 (100%)	Integrating Intimate Partner Violence Screening Programs in Primary Care: Results from a Hybrid-II Implementation-Effectiveness RCT	This stepped-wedge, hybrid-II, implementation-effectiveness, cluster randomized trial investigated implementation and clinical effectiveness outcomes associated with VHA leadership's use of implementation facilitation to roll out IPV screening programs in mixed-sex/gender and shared-space primary care clinics	Health care organization/delivery of care for WV	N/A<br><br>VA
Preventive Health				
Hamilton, 2023[22]<br>40 (100%)	Enhancing Mental and Physical Health of Women Through Engagement and Retention (EMPOWER) 2.0 QUERI: Study Protocol for a Cluster-Randomized Hybrid Type 3 Effectiveness-Implementation Trial	This study aimed to compare the effectiveness of Replicating Effective Practices and Evidence-Based QI on improved access to and rates of engagement in telehealth preventive lifestyle and mental health services	General mental health	N/A<br><br>VA
Marcotte, 2023[23]<br>883 (100%)	Automated Opt-Out vs Opt-In Patient Outreach Strategies for Breast Cancer Screening: A Randomized Clinical Trial	This study evaluated the effect of an opt-out automatic mammography referral strategy compared with an opt-in automated telephone message strategy, on breast cancer screening	Health care organization/delivery of care for WV<br><br>Cancer care	N/A<br><br>VA; NIH/other government
Chronic Medical Conditions				
Markland, 2023[24]<br>286 (100%)	Optimizing Remote Access to Urinary Incontinence Treatments for Women Veterans (PRACTICAL): Study Protocol for a Pragmatic Clinical Trial Comparing Two Virtual Care Options	This pragmatic clinical trial aimed to increase access to behavioral treatment of urinary incontinence for WVs by comparing the effectiveness of two virtual care delivery modalities	N/A	NA<br><br>VA
Vimalananda, 2016[25]<br>481 (15%)	Weight Loss Among Women and Men in the ASPIRE-VA Behavioral Weight Loss Intervention Trial	This study examined weight loss among WV and men veterans in the Aspiring for Lifelong Health (ASPIRE) trial, a small changes weight loss program	Preventative health	General population/not specified<br><br>VA

© 2025 Goldstein KM et al. *JAMA Network Open*.

Author, Year<br>N (% women)	Title	Objective	Secondary focus areas	Target populations<br><br>Funding
Health care organization/delivery of care for WV				
Yano, 2016[26]<br>N/A	Cluster Randomized Trial of a Multilevel Evidence-Based Quality Improvement Approach to Tailoring VA Patient Aligned Care Teams to the Needs of Women Veterans	This study tested an evidence-based QI approach to tailoring PACT to meet WV needs, incorporating comprehensive primary care services and gender-specific care in gender-sensitive environments, accelerating achievement of PACT tenets for women (Womens Health [WH]-PACT)	Access to care/utilization	N/A<br><br>VA

© 2025 Goldstein KM et al. *JAMA Network Open*.

eTable 4. Included Systematic Reviews

Author, Year, N articles	Title	Objective	Secondary focus area(s)	Prioritized populations<br><br>Funding
**General mental health**				
Creech, 2021[27]<br>21	Clinical Complexity in Women Veterans: A Systematic Review of the Recent Evidence on Mental Health and Physical Health Comorbidities	The aim of this systematic review was to evaluate and synthesize research published between 2008 and 2015 and identified in the WV Health Research Evidence Map as related to mental and physical health comorbidities among WVs	Chronic medical conditions	N/A<br><br>VA
Godier-McBard, 2023[28]<br>24	Barriers and Facilitators to Mental Healthcare for Women Veterans: A Scoping Review	This paper aimed to provide a comprehensive up-to-date scoping review of current knowledge regarding the barriers and facilitators experienced by WVs when accessing mental health services	Access to care/utilization<br><br>Health care organization/delivery of care for WV	N/A<br><br>University
Jones, 2017[29]<br>8	The Psychological Health and Well-being Experiences of Female Military Veterans: A Systematic Review of the Qualitative Literature	To determin what is known about the psychological health and well-being experiences of female military veterans	N/A	OEF/OIF/OND<br><br>Not reported
Orshak, 2022[30]<br>8	Interventions for Women Veterans with Mental Health Care Needs: Findings from a Scoping Review	The purpose of this study was to conduct a scoping review of the literature to summarize interventions for WVs with mental health care needs designed in the VA.	Access to care/utilization<br><br>Health care organization/delivery of care for WV	Justice-involved<br><br>University
**Interpersonal Violence**				
Pulverman, 2019[31]<br>6	Military Sexual Trauma and Sexual Health in Women Veterans: A Systematic Review	This paper aimed to systematically review the existing research on the impact of MST on sexual health in WVs	Reproductive health	History of trauma<br><br>VA
Sparrow, 2017[32]<br>13	Systematic Review of Mental Health Disorders and Intimate Partner Violence Victimisation Among Military Populations	This study systematically reviewed extant studies to summarize the literature exploring IPV victimization and specific mental health problems among male and female military personnel (serving and ex-serving)	General mental health	N/A<br><br>Not reported
Wilson, 2018[33]<br>69	The Prevalence of Military Sexual Trauma: A Meta-Analysis	This meta-analysis aimed to obtain a single estimate of the prevalence of MST across methodologies and participant characteristics	N/A	N/A<br><br>Unfunded
**Substance Use**				

© 2025 Goldstein KM et al. *JAMA Network Open*.

Author, Year, N articles	Title	Objective	Secondary focus area(s)	Prioritized populations<br><br>Funding
Simpson, 2022[34]<br>44	Seeking Care Where They Can: A Systematic Review of Global Trends in Online Alcohol Treatment Utilization Among Non-Veteran and Veteran Women	This systematic review examined associations between gender-tailored recruitment/inclusion criteria and proportions of women enrolled in online alcohol intervention trials, evaluated whether community samples have greater proportions of women than clinical samples, and compared country-specific average proportions of women in trials to country-specific proportions of women with AUD	Access to care/utilization	N/A<br><br>Not reported
Weinberger, 2016 [35]	A Review of Research on Smoking Behavior in Three Demographic Groups of Veterans: Women, Racial/Ethnic Minorities, and Sexual Orientation Minorities	This study reviewed published studies of smoking behavior in three demographic subgroups of veterans: women, racial and ethnic minorities, and sexual orientation minorities to synthesize current knowledge and identify areas in need of more research	Preventative health	Sexual minoritized populations; Racial and ethnic minoritized populations<br><br>VA
Chronic Medical Conditions				
Coughlin, 2017[36]<br>21	A Review of Epidemiologic Studies of the Health of Gulf War Women Veterans	This study reviewed epidemiologic studies of the health of women who served in the 1990-1991 Gulf War	General mental health<br>Reproductive health	Gulf War I Veterans<br><br>NIH/other government
Reproductive Health				
Katon, 2018[37]<br>52	Reproductive Health of Women Veterans: A Systematic Review of the Literature from 2008 to 2017	This systematic review aimed to (1) review the literature pertaining to reproductive health of WVs and (2) synthesize findings	N/A	N/A<br><br>VA; NIH/other government
Long-term Care/Aging				
Varilek, 2021[38]<br>19	Female Veteran Use of Palliative and Hospice Care: A Scoping Review	This scoping review explored the palliative and hospice care literature specific to WV to: (1) learn what evidence was available regarding WVs' use of palliative and hospice care, and (2) identify the existing gaps specific to WVs' use of palliative and hospice care, to meet the needs of this growing population	Access to care/utilization<br>Health care organization/delivery of care for WV	N/A<br><br>Not reported
Weitlauf, 2023[39]<br>6	Mortality of Women Vietnam War Era Veterans	The objectives of this study were to 1) understand the scope of the current mortality literature on US Vietnam War Era WVs and 2) identify major themes and knowledge gaps that might guide future research.	Preventative health<br><br>Chronic medical conditions	Vietnam Era<br><br>VA; DOD
Health care Organization/Delivery of care for WVs				
Danan, 2019[40]<br>45	Evidence Map: Reporting of Results by Sex or Gender in Randomized, Controlled Trials with Women Veteran Participants (2008 to 2018)	The main purpose of this study was to compare characteristics of RCTs that included WVs and did or did not report results by sex or gender	NA	NA<br><br>VA
Access to Care/Utilization				

© 2025 Goldstein KM et al. *JAMA Network Open*.

Author, Year, N articles	Title	Objective	Secondary focus area(s)	Prioritized populations<br><br>Funding
Flike, 2023[41]<br>35	Systematic Review of Access to Healthcare and Social Services Among US Women Veterans Experiencing Homelessness	This systematic review examined the barriers and facilitators for access to health care and social care among WVs experiencing homelessness	SDOH	People with experiences of homelessness<br><br>Unfunded
Social Determinants of Health				
Kondo, 2017[42]<br>109	Health Disparities in Veterans: A Map of the Evidence	This study aimed to characterize the research on health care disparities in the Veterans Health Administration via an evidence map	Access to care/utilization<br><br>Health care organization/delivery of care for WV	N/A<br><br>VA
Short, 2023[43]<br>15	Female Veterans' Risk Factors for Homelessness: A Scoping Review	This scoping review aimed to identify published peer-reviewed academic and grey literature about the lived experiences of homeless WVs and risk factors for homelessness among WVs	N/A	People with experiences of homelessness<br><br>Veterans Affairs Canada

© 2025 Goldstein KM et al. *JAMA Network Open*.

REFERENCES

1. Acierno R, Jaffe AE, Gilmore AK, et al. A randomized clinical trial of in-person vs. home-based telemedicine delivery of Prolonged Exposure for PTSD in military sexual trauma survivors. *Journal of Anxiety Disorders*. 2021;83doi:10.1016/j.janxdis.2021.102461
2. Amsalem D, Lazarov A, Markowitz JC, Gorman D, Dixon LB, Neria Y. Increasing treatment-seeking intentions of US veterans in the Covid-19 era: A randomized controlled trial. *Depression & Anxiety (1091-4269)*. 2021;38(6):639-647. doi:10.1002/da.23149
3. Castillo DT, Chee CL, Nason E, et al. Group-Delivered Cognitive/Exposure Therapy for PTSD in Women Veterans: A Randomized Controlled Trial. *Psychological Trauma: Theory, Research, Practice & Policy*. 2016;8(3):404-412. doi:10.1037/tra0000111
4. Creech SK, Pulverman CS, Kahler CW, et al. Computerized Intervention in Primary Care for Women Veterans with Sexual Assault Histories and Psychosocial Health Risks: a Randomized Clinical Trial. *JGIM: Journal of General Internal Medicine*. 2022;37(5):1097-1107. doi:10.1007/s11606-021-06851-0
5. Gobin RL, Strauss JL, Golshan S, et al. Gender Differences in Response to Acceptance and Commitment Therapy Among Operation Enduring Freedom/Operation Iraqi Freedom/Operation New Dawn Veterans. *Women's Health Issues*. 2019;29(3):267-273. doi:10.1016/j.whi.2019.03.003
6. Kelly U, Haywood T, Segell E, Higgins M. Trauma-Sensitive Yoga for Post-Traumatic Stress Disorder in Women Veterans who Experienced Military Sexual Trauma: Interim Results from a Randomized Controlled Trial. *Journal of Alternative & Complementary Medicine*. 2021;27:S-45. doi:10.1089/acm.2020.0417
7. Lehavot K, Millard SP, Thomas RM, et al. A randomized trial of an online, coach-assisted self-management PTSD intervention tailored for women veterans. *J Consult Clin Psychol*. 2021;89(2):134-141. doi:10.1037/ccp0000556
8. Lopez CM, Gilmore AK, Brown WJ, et al. Effects of Emotion Dysregulation on Post-treatment Post-traumatic Stress Disorder and Depressive Symptoms Among Women Veterans With Military Sexual Trauma. *Journal of Interpersonal Violence*. 2022;37(15/16):NP13143-NP13161. doi:10.1177/08862605211005134
9. Martin JL, Carlson GC, Kelly MR, et al. Novel treatment based on acceptance and commitment therapy versus cognitive behavioral therapy for insomnia: A randomized comparative effectiveness trial in women veterans. *J Consult Clin Psychol*. 2023;doi:10.1037/ccp0000836
10. Murdoch M, Clothier BA, Kehle-Forbes S, Vang D, Noorbaloochi S. Impact of different cover letter information and incentives on Veterans' emotional responses to an unsolicited mailed survey about military traumas: a randomized, 3x2x2 factorial trial. *BMC Medical Research Methodology*. 2022;22(1):308-308. doi:10.1186/s12874-022-01783-7
11. Saban KL, Collins EG, Mathews HL, et al. Impact of a Mindfulness-Based Stress Reduction Program on Psychological Well-Being, Cortisol, and Inflammation in Women Veterans. *JGIM: Journal of General Internal Medicine*. 2022;37:751-761. doi:10.1007/s11606-022-07584-4
12. Thompson-Hollands J, Lunney CA, Sloan DM, Wiltsey Stirman S, Schnurr PP. Treatment length and symptom improvement in prolonged exposure and present-centered therapy for posttraumatic stress disorder: Comparing dose-response and good-enough level

© 2025 Goldstein KM et al. *JAMA Network Open*.

models in two manualized interventions. *J Consult Clin Psychol*. 2023;91(10):596-605. doi:10.1037/ccp0000834
13. Zaccari B, Loftis JM, Haywood T, Hubbard K, Clark J, Kelly UA. Synchronous Telehealth Yoga and Cognitive Processing Group Therapies for Women Veterans with Posttraumatic Stress Disorder: A Multisite Randomized Controlled Trial Adapted for COVID-19. *Telemedicine journal and e-health : the official journal of the American Telemedicine Association*. 2022;doi:10.1089/tmj.2021.0612
14. Danan ER, Sherman SE, Clothier BA, et al. Smoking Cessation among Female and Male Veterans before and after a Randomized Trial of Proactive Outreach. *Womens Health Issues*. 2019;29 Suppl 1:S15-S23. doi:https://dx.doi.org/10.1016/j.whi.2019.04.001
15. Holzhauer CG, Epstein EE, Smelson DA, Mattocks K. Targeting women veteran's stress-induced drinking with cognitive reappraisal: Mechanisms and moderators of change. *J Subst Abuse Treat*. 2021;130:N.PAG-N.PAG. doi:10.1016/j.jsat.2021.108408
16. Najavits LM, Enggasser J, Brief D, Federman E. A randomized controlled trial of a gender-focused addiction model versus 12-step facilitation for women veterans. *American Journal on Addictions*. 2018;27(3):210-216. doi:10.1111/ajad.12709
17. Pedersen ER, Parast L, Marshall GN, Schell TL, Neighbors C. A randomized controlled trial of a web-based, personalized normative feedback alcohol intervention for young-adult veterans. *J Consult Clin Psychol*. 2017;85(5):459-470. doi:10.1037/ccp0000187
18. Gilmore AK, Davis MT, Grubaugh A, et al. "Do you expect me to receive PTSD care in a setting where most of the other patients remind me of the perpetrator?": Home-based telemedicine to address barriers to care unique to military sexual trauma and veterans affairs hospitals. *Contemporary Clinical Trials*. 2016;48:59-64. doi:10.1016/j.cct.2016.03.004
19. Gilmore AK, Lopez C, Muzzy W, et al. Emotion Dysregulation Predicts Dropout from Prolonged Exposure Treatment among Women Veterans with Military Sexual Trauma-Related Posttraumatic Stress Disorder. *Women's Health Issues*. 2020;30(6):462-469. doi:10.1016/j.whi.2020.07.004
20. Iverson KM, Danitz SB, Shayani DR, et al. Recovering From Intimate Partner Violence Through Strengths and Empowerment: Findings From a Randomized Clinical Trial. *J Clin Psychiatry*. 2021;83(1):23. doi:https://dx.doi.org/10.4088/JCP.21m14041
21. Iverson KM, Stolzmann KL, Brady JE, et al. Integrating Intimate Partner Violence Screening Programs in Primary Care: Results from a Hybrid-II Implementation-Effectiveness RCT. *American Journal of Preventive Medicine*. 2023;65(2):251-260. doi:10.1016/j.amepre.2023.02.013
22. Hamilton AB, Finley EP, Bean-Mayberry B, et al. Enhancing Mental and Physical Health of Women through Engagement and Retention (EMPOWER) 2.0 QUERI: study protocol for a cluster-randomized hybrid type 3 effectiveness-implementation trial. *Implement*. 2023;4(1):23. doi:https://dx.doi.org/10.1186/s43058-022-00389-w
23. Marcotte LM, Deeds S, Wheat C, et al. Automated Opt-Out vs Opt-In Patient Outreach Strategies for Breast Cancer Screening: A Randomized Clinical Trial. *JAMA Internal Medicine*. 2023;doi:10.1001/jamainternmed.2023.4321
24. Markland AD, Vaughan CP, Goldstein KM, et al. Optimizing remote access to urinary incontinence treatments for women veterans (PRACTICAL): Study protocol for a pragmatic clinical trial comparing two virtual care options. *Contemporary Clinical Trials*. 2023;133doi:10.1016/j.cct.2023.107328

© 2025 Goldstein KM et al. *JAMA Network Open*.

25. Vimalananda V, Damschroder L, Janney CA, et al. Weight loss among women and men in the ASPIRE-VA behavioral weight loss intervention trial. *Obesity (19307381)*. 2016;24(9):1884-1891. doi:10.1002/oby.21574
26. Yano EM, Darling JE, Hamilton AB, et al. Cluster randomized trial of a multilevel evidence-based quality improvement approach to tailoring VA Patient Aligned Care Teams to the needs of women Veterans. *Implementation Science*. 2016;11(1):1-14. doi:10.1186/s13012-016-0461-z
27. Creech SK, Pulverman CS, Crawford JN, et al. Clinical Complexity in Women Veterans: A Systematic Review of the Recent Evidence on Mental Health and Physical Health Comorbidities. *Behavioral Medicine*. 2021;47(1):69-87. doi:10.1080/08964289.2019.1644283
28. Godier-McBard LR, Wood A, Kohomange M, Cable G, Fossey M. Barriers and facilitators to mental healthcare for women veterans: a scoping review. *Journal of Mental Health*. 2023;32(5):951-961. doi:10.1080/09638237.2022.2118686
29. Jones GL, Hanley T. The psychological health and well-being experiences of female military veterans: a systematic review of the qualitative literature. *Journal of the Royal Army Medical Corps*. 2017;163(5):311-318. doi:10.1136/jramc-2016-000705
30. Orshak J, Alexander L, Gilmore-Bykovskyi A, Lauver D. Interventions for Women Veterans with Mental Health Care Needs: Findings from a Scoping Review. *Issues in mental health nursing*. 2022;43(6):516-527. doi:10.1080/01612840.2021.2011506
31. Pulverman CS, Christy AY, Kelly UA. Military Sexual Trauma and Sexual Health in Women Veterans: A Systematic Review. *Sexual Medicine Reviews*. 2019;7(3):393-407. doi:10.1016/j.sxmr.2019.03.002
32. Sparrow K, Kwan J, Howard L, Fear N, MacManus D. Systematic review of mental health disorders and intimate partner violence victimisation among military populations. *Social Psychiatry & Psychiatric Epidemiology*. 2017;52(9):1059-1080. doi:10.1007/s00127-017-1423-8
33. Wilson LC. The Prevalence of Military Sexual Trauma: A Meta-Analysis. *Trauma, Violence & Abuse*. 2018;19(5):584-597. doi:10.1177/1524838016683459
34. Simpson T, Sistad R, Brooks JT, Newberger NG, Livingston NA. Seeking care where they can: A systematic review of global trends in online alcohol treatment utilization among non-veteran and veteran women. *Drug and Alcohol Dependence Reports*. 2022;5doi:10.1016/j.dadr.2022.100116
35. Weinberger AH, Esan H, Hunt MG, Hoff RA. A review of research on smoking behavior in three demographic groups of veterans: women, racial/ethnic minorities, and sexual orientation minorities. *Am J Drug Alcohol Abuse*. 2016;42(3):254-268. doi:10.3109/00952990.2015.1045978
36. Coughlin SS, Krengel M, Sullivan K, Pierce PF, Heboyan V, Wilson LCC. A Review of Epidemiologic Studies of the Health of Gulf War Women Veterans. *J*. 2017;3(2)doi:https://dx.doi.org/10.15436/2378-6841.17.1551
37. Katon JG, Zephyrin L, Meoli A, et al. Reproductive Health of Women Veterans: A Systematic Review of the Literature from 2008 to 2017. *Seminars in Reproductive Medicine*. 2018;36(6):315-322. doi:10.1055/s-0039-1678750
38. Varilek BM, Isaacson MJ. Female Veteran Use of Palliative and Hospice Care: A Scoping Review. *Military Medicine*. 2021;186(11/12):1100-1105. doi:10.1093/milmed/usab005
39. Weitlauf JC, Cypel YS, Davey VJ. Mortality of Women Vietnam War–Era Veterans. *Women's Health Issues*. 2023;33(4):391-404. doi:10.1016/j.whi.2023.02.004

© 2025 Goldstein KM et al. *JAMA Network Open*.

40. Danan ER, Ullman K, Klap RS, Yano EM, Krebs EE. Evidence Map: Reporting of Results by Sex or Gender in Randomized, Controlled Trials with Women Veteran Participants (2008 to 2018). *Women's Health Issues*. 2019;29:S112-S120. doi:10.1016/j.whi.2019.04.011
41. Flike K, Byrne T. Systematic review of access to healthcare and social services among US women Veterans experiencing homelessness. *Women's Health (17455057)*. 2023:1-17. doi:10.1177/17455057231189550
42. Kondo K, Low A, Everson T, et al. Health Disparities in Veterans: A Map of the Evidence. *Med Care*. 2017;55 Suppl 9 Suppl 2:S9-S15. doi:https://dx.doi.org/10.1097/MLR.0000000000000756
43. Short M, Felder S, Garland Baird L, Gamble B. Female Veterans' risk factors for homelessness: A scoping review. *Journal of Military, Veteran & Family Health*. 2023;9(4):29-38. doi:10.3138/jmvfh-2022-0069

© 2025 Goldstein KM et al. *JAMA Network Open*.
