# Supplementary material for: An Evidence Map of the Women Veterans’ Health Literature, 2016 to 2023: A Systematic Review
Source: JAMA Netw Open. 2025 Apr 22;8(4):e256372. doi: 10.1001/jamanetworkopen.2025.6372 (PMC12015682; doi:10.1001/jamanetworkopen.2025.6372)
Supplement: Supplement 2. — Data Sharing Statement [file jamanetwopen-e256372-s002.pdf]

Data Sharing Statement

Goldstein. An Evidence Map of the Women Veterans' Health Literature, 2016 to 2023. *JAMA Netw Open*. Published April 22, 2025. doi:10.1001/jamanetworkopen.2025.6372

Data
Data available: Yes
Data types: Other (please specify)
Additional Information: Summary data file of extracted data.
How to access data: karen.goldstein@va.gov
When available: With publication

Supporting Documents
Document types: None

Additional Information
Who can access the data: Data will be made available upon reasonable request.
Types of analyses: For any purpose.
Mechanisms of data availability: Data will be made available according to VA sharing requirements.
